# Design principles for natural SGLT2 modulators from multi-scale computation

**DOI:** 10.1098/rsos.261760

**Published:** 2026-11-04

**Authors:** Oussama Khibech, Said Abadi, Mohamed Bouhrim, Yousra Hammouti, Mohammed Merzouki, Mohamed Addi, Mohammed AL-Zharani, Fahd A. Nasr, Ashraf Ahmed Qurtam, Allal Challioui

**Affiliations:** ^1^ University Mohammed Premier, Faculty of Science, Laboratory of Applied Chemistry and Environment (LCAE), Oujda, 60000 Morocco; ^2^ Laboratoires TBC, Laboratory of Pharmacology, Pharmacokinetics, and Clinical Pharmacy, Faculty of Pharmaceutical and Biological Sciences, Lille, France; ^3^ Laboratoire d'Amélioration des Productions Agricoles, Biotechnologie et Environnement (LAPABE), Faculté des Sciences, Université Mohammed Premier, Oujda 60000, Morocco; ^4^ Biology Department, College of Science, Imam Mohammad Ibn Saud Islamic University (IMSIU), Riyadh, 11623, Saudi Arabia

**Keywords:** SGLT2, natural products, membrane molecular dynamics, MM/PBSA, principal component analysis, DFT

## Abstract

Human sodium-glucose cotransporter 2 (hSGLT2) is a membrane-embedded renal transporter and an established therapeutic target for type 2 diabetes. Hyperoside, myricetin, cyanidin-3-O-glucoside and isoliquiritigenin were investigated as a chemically diverse panel of natural scaffolds using a multi-scale computational workflow combining redocking-validated molecular docking, reconstruction of an unresolved transporter segment, molecular dynamics (MD) in an explicit POPC bilayer, molecular mechanics/Poisson–Boltzmann surface area (MM/PBSA) calculations, principal component and free-energy landscape analyses and density functional theory (DFT). The membrane simulations preserved the global hSGLT2 fold while revealing distinct ligand-dependent binding behaviours. Myricetin and cyanidin-3-O-glucoside exhibited limited positional deviations; hyperoside achieved persistent accommodation following an initial adjustment, whereas isoliquiritigenin underwent a marked late rearrangement. Ensemble-based energetic analysis prioritized cyanidin-3-O-glucoside, while hyperoside and myricetin formed an energetically comparable intermediate group and isoliquiritigenin displayed the least favourable profile. Electronic-structure analysis further characterized cyanidin-3-O-glucoside as the most responsive ligand within the DFT subset. Collectively, these findings indicate that glycone-assisted polar anchoring, aromatic complementarity and restricted conformational flexibility promote persistent hSGLT2 recognition. Direct hSGLT2 inhibition and hSGLT1 counter-screening assays remain necessary to establish potency and selectivity.

## 1. Introduction

The human sodium-glucose cotransporter 2 (hSGLT2) is an apical membrane symporter of the renal proximal tubule that couples the inward Na⁺ electrochemical gradient to glucose uptake, thereby reclaiming most filtered glucose and limiting urinary glucose loss; pharmacological interruption of this transport cycle underlies the clinical success and cardiorenal benefits of gliflozins [1–4]. At the molecular level, the SGLT2 binding cavity is organized around a polar sugar-recognition cradle flanked by hydrophobic and aromatic subsites, thereby favouring ligands that can satisfy a stringent combination of directional hydrogen bonding, *π*-surface complementarity and steric fit [5,6]. This architecture is especially relevant to natural-product-inspired ligand design because the field has historically emerged from phlorizin and natural flavonoids; glycosides and related polyphenols continue to inform SGLT2 medicinal chemistry and transporter pharmacology [4,7].

Within this structural framework, the present study examines four natural chemotypes selected as a deliberately orthogonal mechanistic panel rather than as a common-scaffold series or arbitrary phytochemical collection. Hyperoside (quercetin-3-O-galactoside) and cyanidin-3-O-glucoside probes glycone-assisted recognition through dense hydrogen-bonding interactions with the polar cradle while preserving extended aromatic surfaces for hydrophobic and *π*-stacking contacts; at the same time, they represent electronically distinct flavonol and anthocyanin chromophores [8–10]. Myricetin, the corresponding flavonol aglycone, removes the sugar moiety and thus isolates the contribution of the polyhydroxylated aromatic core to the insertion depth, *π*-stacking behaviour and the balance between donor and acceptor interactions [11–13]. Isoliquiritigenin, a chalcone bearing an *α*,*β*-unsaturated carbonyl linker, introduces greater conformational adaptability together with a different distribution of lone pairs and *π*-density, enabling a mechanistically distinct mode of subsite bridging [14–17]. Collectively, the set isolates the effects of glycosylation, hydroxylation pattern, chromophore electronics, conjugation and scaffold flexibility, thereby enabling a non-redundant mechanism-driven comparison within the SGLT2 pocket.

Methodologically, docking alone can overestimate affinity and cannot fully capture solvent competition, conformational plasticity, local gating or entropic penalties—limitations that are particularly important for membrane transporters, in which lipid coupling, hydration networks and side-chain rearrangements influence ligand recognition and residence [18–21]. Although previous computational studies on SGLT2 have provided useful screening, classification and structure-based optimization frameworks, comparatively few have examined a deliberately selected set of chemically distinct natural scaffolds within a re-docking-validated, membrane-embedded multi-scale workflow [22]. As a result, the field still lacks a coherent mechanism-oriented picture linking pocket recognition, dynamic persistence, ensemble energetics, collective conformational behaviour and ligand-intrinsic electronic structure for a focused natural series.

To address this gap, we integrated validated molecular docking, 100 ns molecular dynamics (MD) simulations in an explicit POPC bilayer, molecular mechanics/Poisson–Boltzmann surface area (MM/PBSA) binding-energy analysis, principal component/free-energy landscape analysis and density functional theory (DFT) to establish a traceable bridge from atomic contacts to electronic propensity [23–28]. In addition to frontier-orbital descriptors and gap-derived reactivity metrics, DFT-based real-space analyses, including molecular electrostatic potential (MEP), electron localization function (ELF) and localized orbital locator (LOL), were used to map donor/acceptor hot spots, *π*-delocalization pathways and electron-pair localization across the four ligands. This integrated strategy goes beyond simply ranking natural candidates; instead, it explains why certain scaffolds remain stably engaged within the SGLT2 binding cavity. These computational predictions were further interpreted in light of previously reported biochemical and anti-diabetic evidence [7,29–32], with the aim of delivering electronically rationalized leads and experimentally testable design principles for natural SGLT2 modulation.

## 2. Materials and methods

### 2.1. Prediction of the toxicity characteristics

Prospective toxicity profiling was performed using the ProTox-III web platform [33] following the recommended workflow. Canonical SMILES strings of the four ligands (shown in scheme 1) were generated in ChemDraw, validated for valence and charge consistency and submitted for prediction. ProTox-III applies statistical modelling and machine learning classifiers trained on curated toxicology datasets to estimate multiple endpoints, including LD_50_-based acute toxicity and globally harmonized system (GHS) classifications [34]. The resulting *in silico* profiles supported rational compound prioritization for subsequent experiments and enabled data-driven decision-making in pharmaceutical development and environmental risk assessment.

**Scheme 1.**
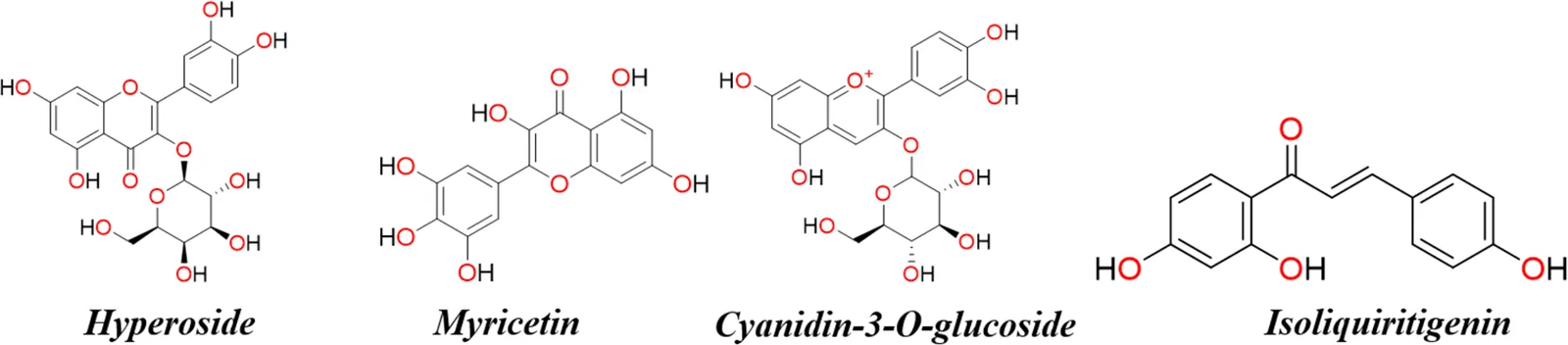
Chemical structures of the four ligands investigated in this study: hyperoside, myricetin, cyanidin-3-O-glucoside and isoliquiritigenin.

### 2.2. PyRx: preparation, configuration and validation of the docking protocol

The hSGLT2 structure in complex with MAP17 (PDB ID: 7VSI, cryo-EM, 2.95 Å) was downloaded from the Protein Data Bank. The stereochemical quality was checked by Ramachandran analysis, which showed 89.4% of residues in the most favoured regions and 10.6% in additionally allowed regions, with 0.0% in the disallowed regions (figure 1). Receptor preparation was performed in AutoDock Tools v1.5.7 by removing crystallographic water molecules and non-essential heteroatoms, adding polar hydrogens, assigning Gasteiger partial charges and exporting the rigid protein as PDBQT files.

**Figure 1.**
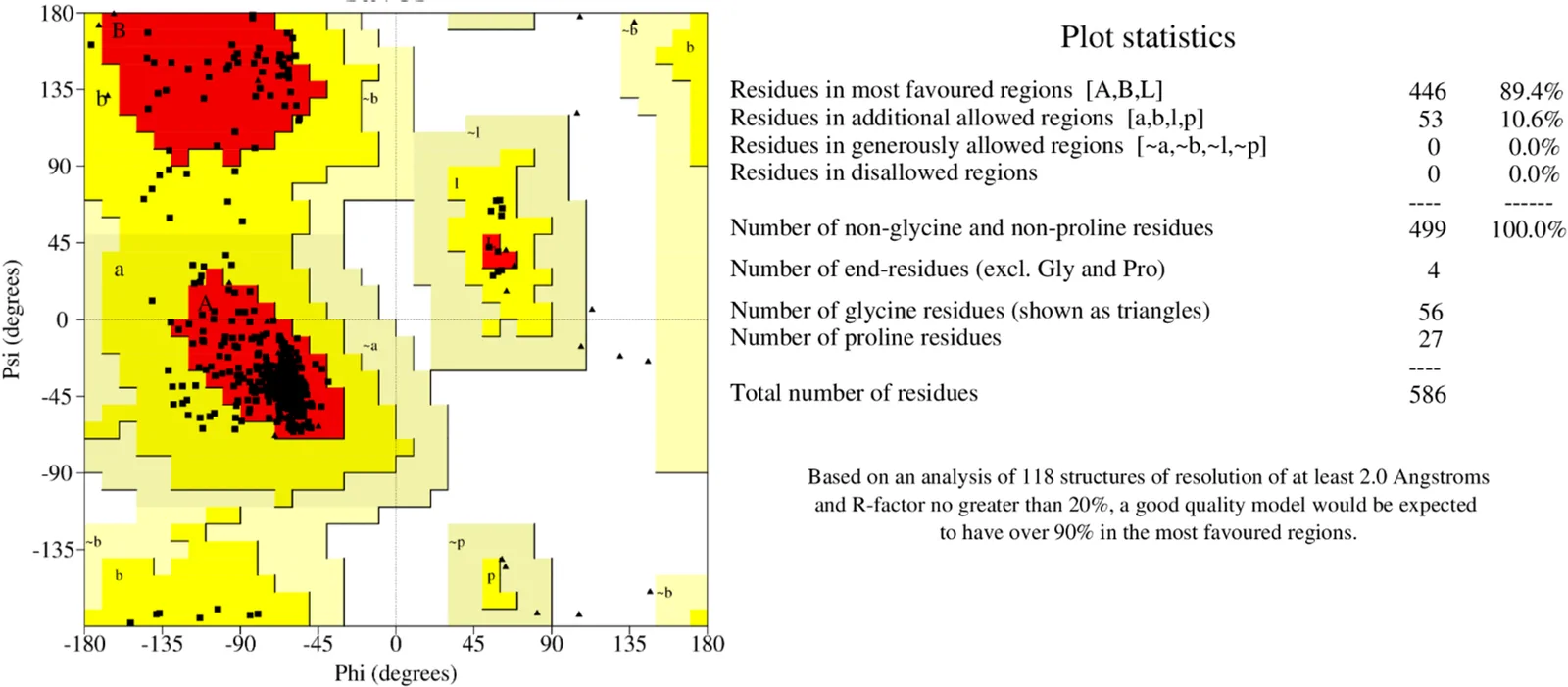
Ramachandran quality assessment of hSGLT2 (PDB 7VSI).

Four natural ligands—hyperoside, myricetin, cyanidin-3-O-glucoside (flavylium form) and isoliquiritigenin—were retrieved from PubChem, converted to three-dimensional structures and energy-minimized using Open Babel through PyRx v0.9.9 (default settings). The ligands were protonated for physiological pH when applicable and saved as flexible PDBQT files with Gasteiger charges.

Targeted docking was carried out with AutoDock Vina (as bundled in PyRx v0.9.9; Vina 1.2.0) using a grid box centred on the co-crystallized ligand site with the following parameters: centre^x^ = 39.32 Å, centre^y^ = 51.35 Å and centre^z^ = 45.00 Å; size^x^ = 12.14 Å, size^y^ = 15.33 Å and size^z^ = 15.53 Å; and exhaustiveness = 8 [35]. For each ligand, Vina generated alternative poses ranked by predicted binding affinity (kcal mol^−1^). The top-scoring pose that also satisfied steric and interaction plausibility within the binding pocket was retained after visual inspection.

Protocol validation was performed by re-docking the co-crystallized inhibitor empagliflozin into the 7VSI structure, which yielded a heavy-atom root mean square deviation (RMSD) of 0.998 Å between the predicted and experimental poses (figure 2). This confirmed that the search space and docking parameters were appropriate for exploring ligand recognition at the SGLT2 binding site.

**Figure 2.**
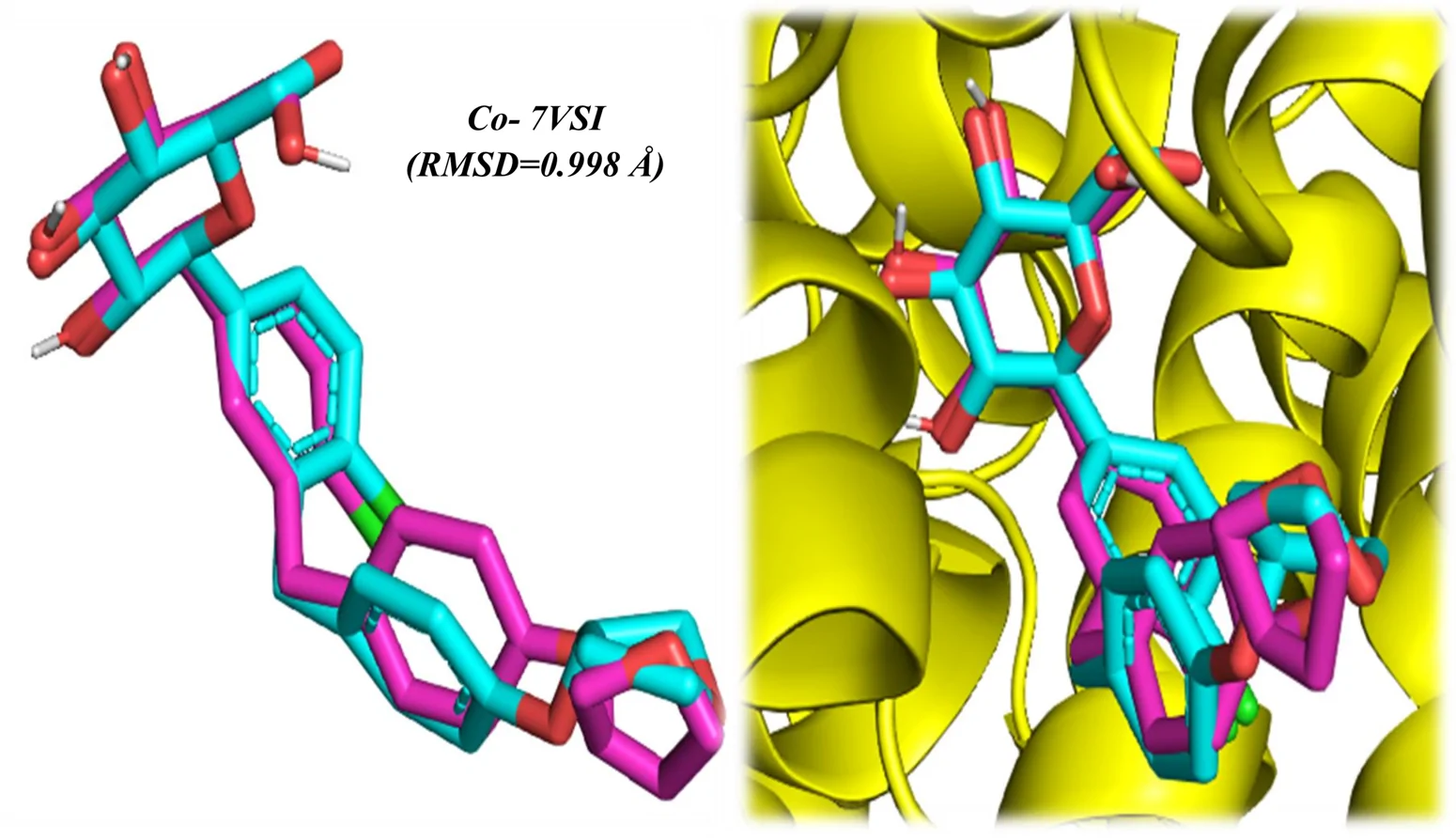
Redocking validation of the docking protocol for SGLT2 (PDB 7VSI; heavy-atom RMSD = 0.998 Å).

Molecular graphics, overlays and binding-site depictions were generated in PyMOL v2.5 (Schrödinger, LLC) and Discovery Studio Visualizer 2024. Interaction maps and two-dimensional contact diagrams were produced using Discovery Studio Visualizer 2024, whereas three-dimensional superpositions and pocket renderings were created using PyMOL.

### 2.3. Implementation of molecular dynamics simulations using GROMACS

The experimentally unresolved protein segment spanning residues 571–641 was reconstructed using MODELLER v10.8 through a hierarchical, two-stage modelling protocol. In the first stage, 100 comparative models were generated using AutoModel and ranked according to their discrete optimized protein energy (DOPE) and GA341 scores, steric-clash profiles, backbone geometry and energy-minimization behaviour. The highest-ranked candidate was subsequently evaluated using PROCHECK within SAVES v6.1, which revealed residual Ramachandran outliers localized within the residues 625–641. This interval was therefore defined as a targeted refinement window rather than an additional missing segment; residues extending beyond position 641 were included as flanking residues to reduce boundary strain and improve local backbone geometry. A second ensemble of 100 loop-refined models was generated for residues 625–641 using the MODELLER LoopModel protocol and ranked primarily according to the local DOPE score of the refined region and secondarily according to the global DOPE score, followed by renewed stereochemical validation using SAVES v6.1. The final model was selected based on the optimal combination of energetic quality, Ramachandran statistics and overall stereochemical reliability. As shown in figure 3A, the reconstructed region was located more than 20 Å from the active-site pocket and did not meaningfully affect the predicted binding region; consequently, the experimentally resolved receptor structure was used for molecular docking, whereas the completed and stereochemically refined structure was used for membrane-system construction and MD simulations. The selected protein–ligand complex was embedded in a POPC lipid bilayer using CHARMM-GUI Membrane Builder and parameterized using the CHARMM36m protein force field together with CHARMM36 lipid parameters. The system was solvated with CHARMM-modified TIP3P water and neutralized with counterions, as illustrated in figure 3B. All simulations were performed using GROMACS 2023.3 under periodic boundary conditions. Energy minimization was conducted using the steepest-descent algorithm for a maximum of 5000 steps, with a convergence threshold of 1000 kJ mol^−1^ nm^−1^, followed by six sequential restrained equilibration stages totalling 1.875 ns, comprising 250 ps under NVT conditions and 1.625 ns under semi-isotropic NPT conditions, with progressive release of protein and lipid restraints. An unrestrained 100 ns production trajectory was subsequently generated and analysed at 310 K and 1 bar using a 2 fs integration time step, a V-rescale thermostat independently applied to the solute, membrane, solvent groups (*τ*_t_ = 1.0 ps) and a semi-isotropic C-rescale barostat (*τ*_p_ = 5.0 ps; compressibility = 4.5 × 10^−5^ bar^−1^). Non-bonded interactions were treated using the Verlet neighbour-list scheme with nstlist = 20 and rlist = 1.2 nm. Lennard–Jones interactions were force-switched between 1.0 and 1.2 nm. Long-range electrostatic interactions were calculated using the particle-mesh Ewald method with a real-space cut-off of 1.2 nm. Bonds involving hydrogen atoms were constrained using the LINCS algorithm. The resulting trajectory was used to assess the conformational stability of the membrane-embedded complex and the persistence of protein–ligand interactions [36].

**Figure 3.**
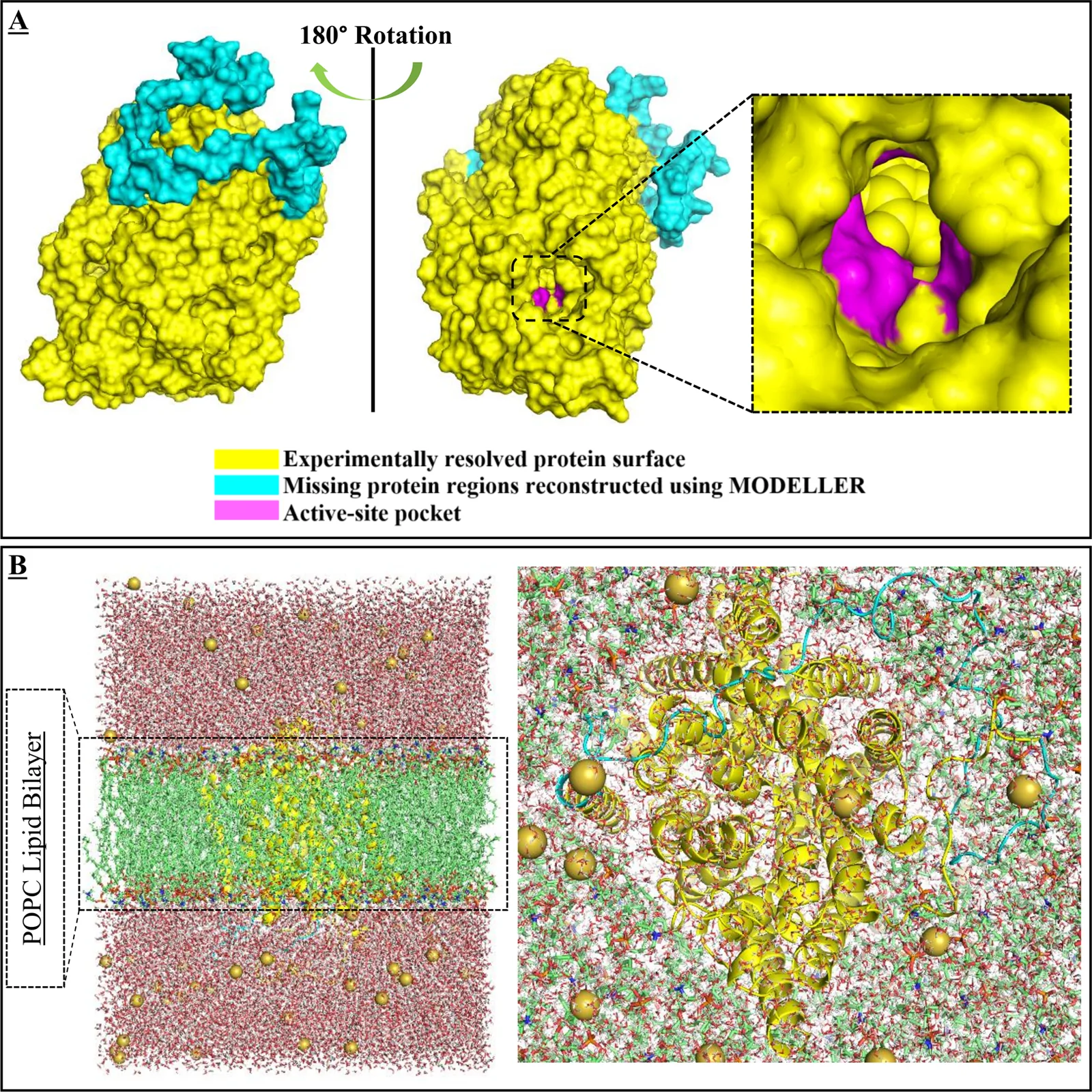
Reconstruction of unresolved hSGLT2 regions and membrane-system set-up. (A) Surface representation of hSGLT2 showing the MODELLER-reconstructed regions in cyan, the experimentally resolved structure in yellow and the active-site pocket in magenta. The reconstructed segment was located more than 20 Å from the binding site. (B) Protein–ligand complex embedded in a POPC bilayer and solvated with TIP3P water and ions using CHARMM-GUI, shown from the side and membrane-normal views.

### 2.4. Molecular mechanics/Poisson–Boltzmann surface area binding-energy calculations

MM/PBSA binding-energy calculations were performed using gmx_MMPBSA v1.6.5 coupled with AmberTools 23 [37]. A total of 100 uniformly distributed snapshots were extracted from the equilibrated 75–100 ns interval of each trajectory and analysed using the single-trajectory approach. For the end-state calculations, the receptor–ligand complex was extracted from each snapshot, while explicit POPC lipids, water molecules and ions were excluded. The polar solvation contribution was estimated using the Poisson–Boltzmann model with dielectric constants of *ε*_in_ = 1.0 and *ε*_out_ = 80.0 and an ionic strength of 0.15 M, whereas the non-polar solvation contribution was calculated from the solvent-accessible surface area. Because neither the heterogeneous membrane dielectric environment nor the configurational entropy was explicitly considered, the calculated energies were interpreted exclusively as relative MM/PBSA binding-energy estimates for comparing complexes evaluated under identical conditions, rather than as absolute binding free energies.

### 2.5. Density functional theory methods and surface descriptors

All quantum-chemical calculations were performed in ORCA 5.0.4 for each ligand in its neutral singlet state (q = 0, multiplicity = 1). Ground-state geometries were optimized in the gas phase at B3LYP-D3(BJ)/def2-SVP, using RIJCOSX with the def2/J auxiliary basis, TightSCF convergence and an integration Grid5. A harmonic frequency calculation confirmed true minima (no imaginary frequencies). Frontier molecular orbitals and their energies (E_HOMO_, E_LUMO_) were taken from the converged self-consistent field wavefunction on the optimized structures and conceptual-DFT metrics were derived as Egap = E_LUMO_ - E_HOMO_, *η* = Egap/2, S = 1/(2*η*), *μ* = (E_HOMO_ + E_LUMO_)/2 and *ω* = *μ*^2^/(2*η*).

Real-space analyses were carried out with Multiwfn 3.8 by reading the ORCA wavefunction (Molden format). The ELF and LOL were computed on a fine three-dimensional grid and reported on the canonical 0–1 scale. The MEP was computed from the same wavefunction and mapped onto the electron-density isosurface ρ = 0.001 arb. units (isovalue specified in Multiwfn) and then visualized in Avogadro 1.2.0. MEP values are reported in eV (conversion 1 arb. units = 27.2114 eV); identical colour maps and fixed display parameters (isosurface levels, camera and lighting) were enforced across the ligands to ensure reproducibility and cross-comparison. Highest occupied molecular orbital and lowest unoccupied molecular orbital (HOMO and LUMO) isosurfaces were rendered in Avogadro at 0.03 arb. units with standard phase colouring. All visualization/analysis settings were kept constant across the figures to guarantee comparability with the MD, MM/PBSA and principal component analysis (PCA) results.

## 3. Results and discussion

### 3.1. *In silico* toxicity assessment and risk-mitigation profiling

*In silico* toxicity assessment is a decisive step prior to docking, MD and candidate prioritization because it anticipates major causes of attrition (systemic toxicity, mechanism-based alerts and CYP interactions) and guides optimization chemistry, dose selection and formulation strategy from the outset. By combining a dose-response view (GHS classes and LD_50_ in figure 4) with a mechanistic, multi-target interaction-probability profile (figure 5), one obtains a coherent picture of the safety margin and structure-specific risks for each compound [36,38].

**Figure 4.**
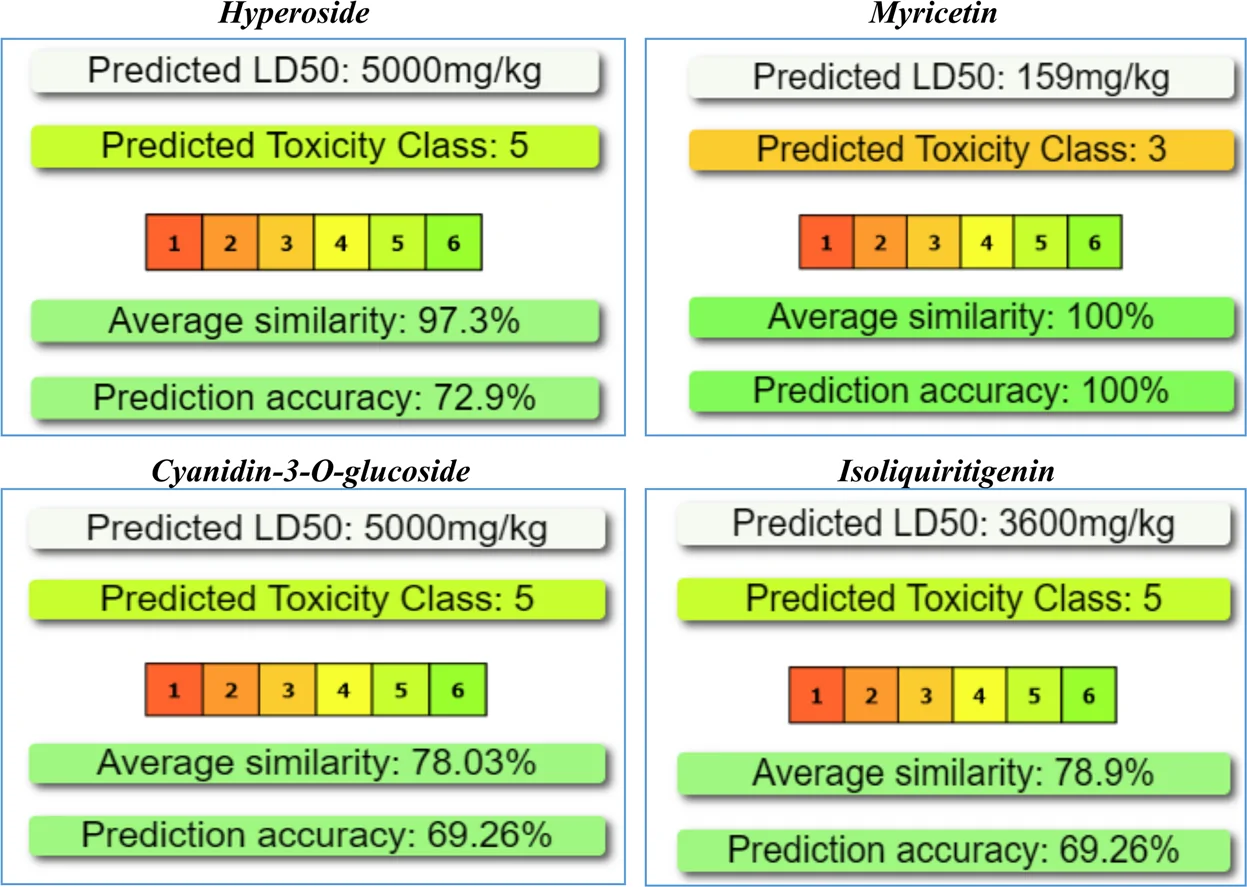
Acute oral toxicity and GHS classification (ProTox-III) for the four SGLT2 ligands.

**Figure 5.**
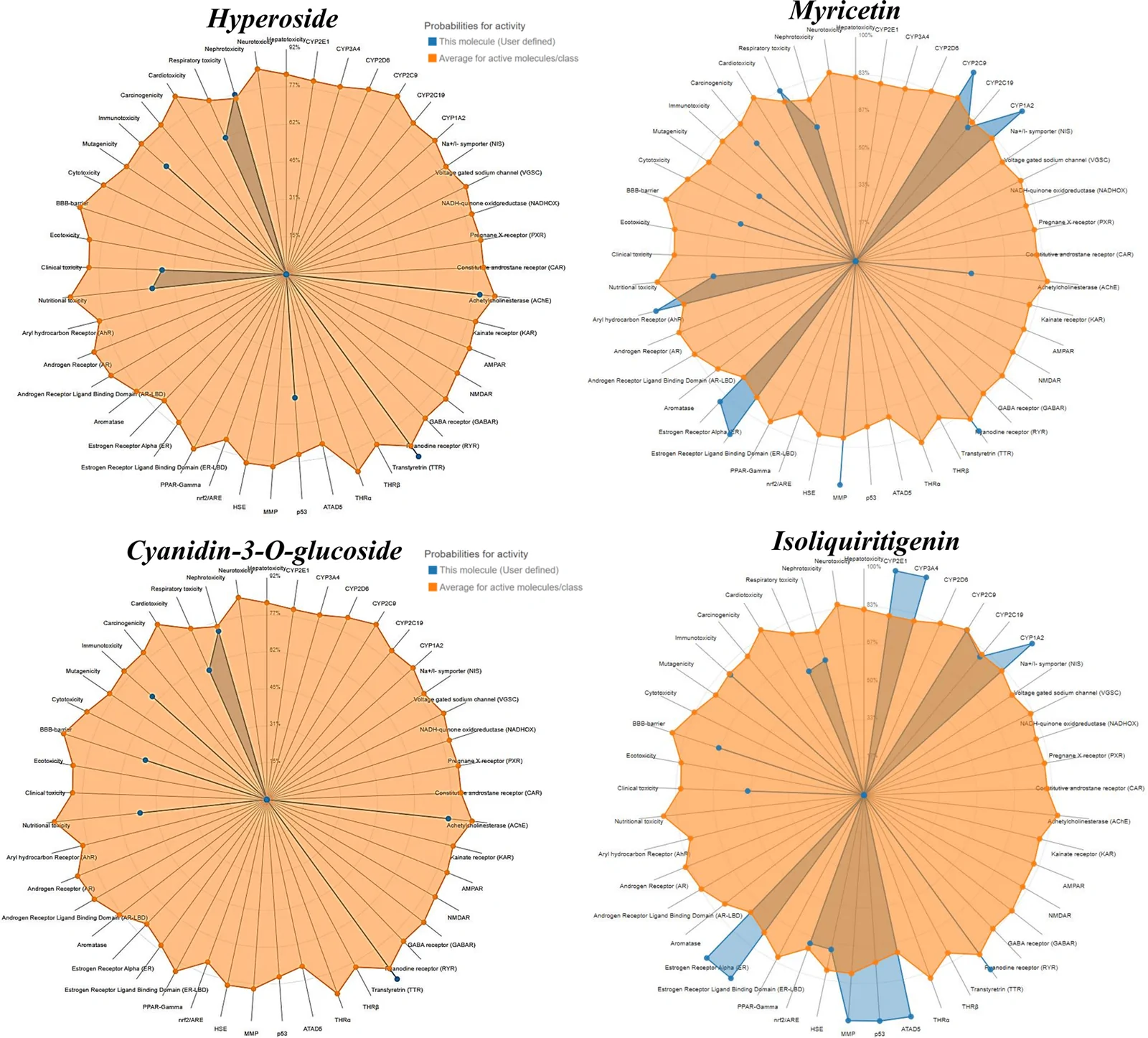
Mechanistic toxicity radar profiles across off-targets and toxicological pathways for the four ligands.

Taken together, the results in figures 4 and 5 show that the two glycosides have the best tolerability, whereas reactive or highly planar aglycones concentrate the alerts. Hyperoside (GHS class 5, LD_50_ ≈ 5000 mg kg^–1^; similarity ≈ 97%, prediction accuracy ≈ 73%) displayed a generally low radar profile with two signals to watch: nephrotoxicity ≈ 77% and transthyretin (TTR) interaction ≈ 92%. These findings are consistent with its high polarity and 3-O-glycoside, which may increase proximal tubular exposure via transporters and with an aromatic surface that can complement the thyroxine channel of TTR [39,40]. Cyanidin-3-O-glucoside (class 5, LD_50_ ≈ 5000 mg kg^–1^; similarity ≈ 78%, accuracy ≈ 69%) showed only a single elevation, TTR ≈ 92%, which is explained by its planar, cationic flavylium core that fits aromatic cavities, while its high hydrophilicity limits other off-target engagements. By contrast, myricetin (class 3, LD_50_ ≈ 159 mg kg^–1^; similarity and accuracy ≈ 100%) markedly exceeded the reference zone for RYR, CYP1A2, CYP2C9, respiratory toxicity, AhR, ER/ER-LBD and MMP. This convergence is expected for a planar flavonol with a B-ring pyrogallol motif that undergoes redox cycling and quinone formation, depletes glutathione and binds promiscuously in CYP heme pockets and nuclear-receptor cavities. Finally, isoliquiritigenin (class 5, LD_50_ ≈ 3600 mg kg^–1^; similarity ≈ 79%, accuracy ≈ 69%) accumulates nine high-probability liabilities near 100% (CYP2E1, CYP3A4, CYP1A2, RYR, ATD5, p53, MMP, ER, ER-LBD), consistent with a chalcone bearing an *α*,*β*-unsaturated enone that acts as a Michael acceptor and is flexible and lipophilic, reacts with thiols and perturbs mitochondrial and endocrine function [41].

These profiles suggest concrete and immediately actionable mitigation levers. For hyperoside and cyanidin-3-O-glucoside, retain the sugar footprint while reducing TTR engagement, favour C-glycosylation (less enzymatic cleavage and renal recirculation), introduce steric bulk at C-3/C-7 or selective O-methylation on the B ring to weaken aromatic stacking and consider luminal kidney-targeted vectors or ‘soft’ prodrugs that hydrolyze rapidly to limit tubular accumulation. For myricetin, decrease planarity and redox propensity by C2-C3 saturation (dihydroflavonol), add 3-O- or C-glycosylation to increase polarity and protect the B-ring triol by O-methylation/acylation to reduce quinone formation and engagement of CYP/AhR/ER [42]. For isoliquiritigenin, attenuate enone electrophilicity by hydrogenation to a dihydrochalcone, cyclization to a more rigid flavanone or prodrug masking (carbonates/phosphates/oximes), while raising polarity through glycosylation to lower CYP and ER affinity. In parallel, early targeted *in vitro* assays (TTR competition, CYP inhibition panels, thiol reactivity and MMP/RYR tests) will confirm the signals in figure 5 and refine selection of the safest derivatives highlighted in figure 4.

### 3.2. Molecular docking

Molecular docking is the first structure-guided filter in this work because it quantifies how well each phytochemical can engage the hSGLT2 (hSGLT2; gene SLC5A2, PDB 7VSI) at atomic resolution and thereby prioritizes chemotypes for the more expensive MD and MM/PBSA stages [43].

SGLT2 is a high-capacity, low-affinity Na⁺/glucose symporter on the apical membrane of proximal-tubule cells that reabsorbs the majority of filtered glucose; its inhibition drives glucosuria and underlies the cardio-renal benefits of gliflozins [44]. The binding pocket combines a polar sugar-recognition cradle with adjacent hydrophobic/aromatic subsites, a topology that naturally discriminates scaffolds by their ability to form directional H-bond networks while positioning a hydrophobic core for *π*-stacking.

The docking results in table 1, together with the pocket overlay in figure 6, indicate that all four phytochemicals occupy the canonical SGLT2 recognition cavity that hosts the co-crystallized inhibitor, engaging the polar sugar-recognition region and the adjacent aromatic/hydrophobic subpockets in a recurrent manner. Among the naturals, hyperoside showed the most favourable predicted affinity (–10.0 kcal mol^–^¹), driven by multidentate hydrogen bonding from its 3-O-glucoside combined with productive placement of the chromophore in the apolar channel, which mirrors the pharmacophore principles of gliflozins [45]. Myricetin (–9.7 kcal mol^–^¹) relies on a mix of short hydrogen bonds and *π*-*π* contacts but, as an aglycone, it lacks the sugar-based chelation that maximizes anchoring in the polar cradle, hence it has an intermediate score. Cyanidin-3-O-glucoside (–8.9 kcal mol^–^¹) uses its glycone to establish directional hydrogen bonds while the flavylium core packs against aromatic surfaces, yielding a mixed polar-aromatic recognition mode. Isoliquiritigenin (–8.9 kcal mol^–^¹) formed fewer polar contacts and depended mainly on *π*-stacking; its open-chain enone reduced shape complementarity relative to the glycosides. Overall, table 1 captures a coherent affinity hierarchy dominated by sugar-driven hydrogen-bonding and aromatic fit and figure 6 confirms co-localization of all poses within the same binding pocket. Detailed residue-level interactions are reserved for figures 7–10.

**Table 1.** Docking scores (AutoDock Vina) and key protein–ligand contacts for four natural ligands at the SGLT2 pocket (PDB 7VSI), compared with the co-crystallized inhibitor (empagliflozin).

	affinity (kcal mol^−1^)			
compounds	7VSI	interactions	bond distances (Å)	H-bond distances (Å)
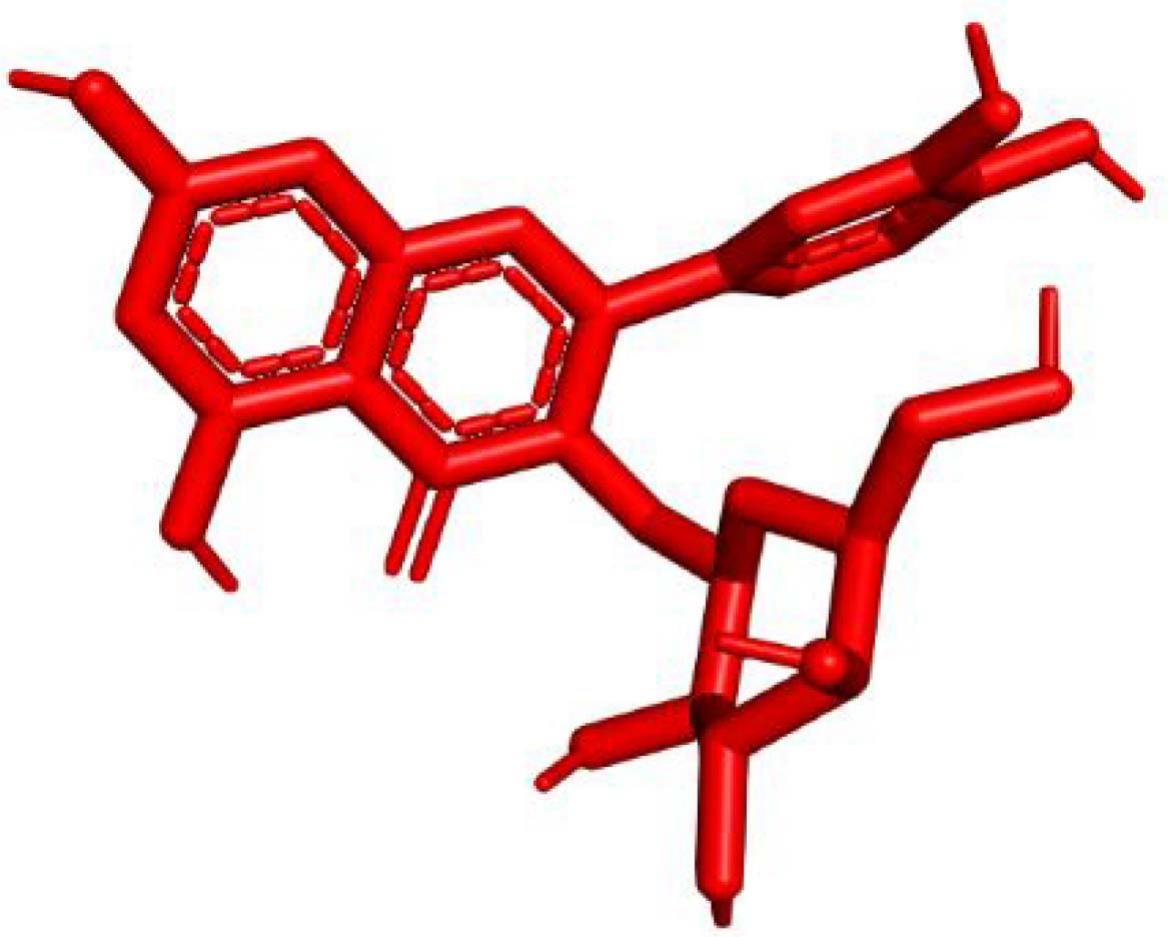 **hyperoside**	−10.0	Leu84 Phe98 Glu99 Ala102 Thr153 Ser287 Tyr290 Trp291 Gln457 Ser460	4.93 2.57; 5.59 4.26 5.19 2.25; 2.48 2.32 3.81 1.97 3.03; 3.37; 4.14 2.70	*** 2.57 *** *** 2.25; 2.48 2.32 *** *** 3.03; 3.37; 4.14 2.70
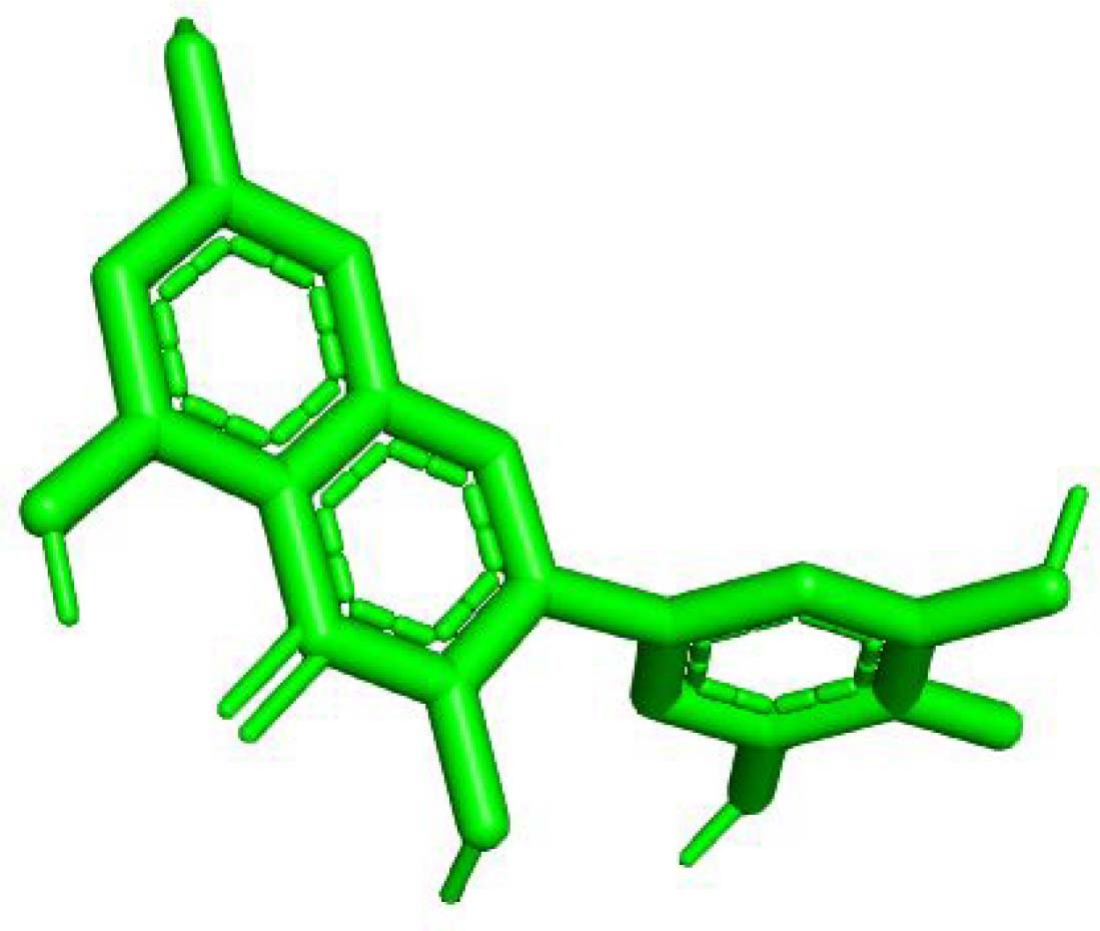 **myricetin**	−9.7	Asn75 His80 Leu84 Phe98 Tyr290 Lys321 Gln457	1.89 3.05; 4.19; 5.10 3.92 5.91 2.94; 4.79 2.67 4.05	1.89 3.05 *** 5.91 2.94 *** 4.05
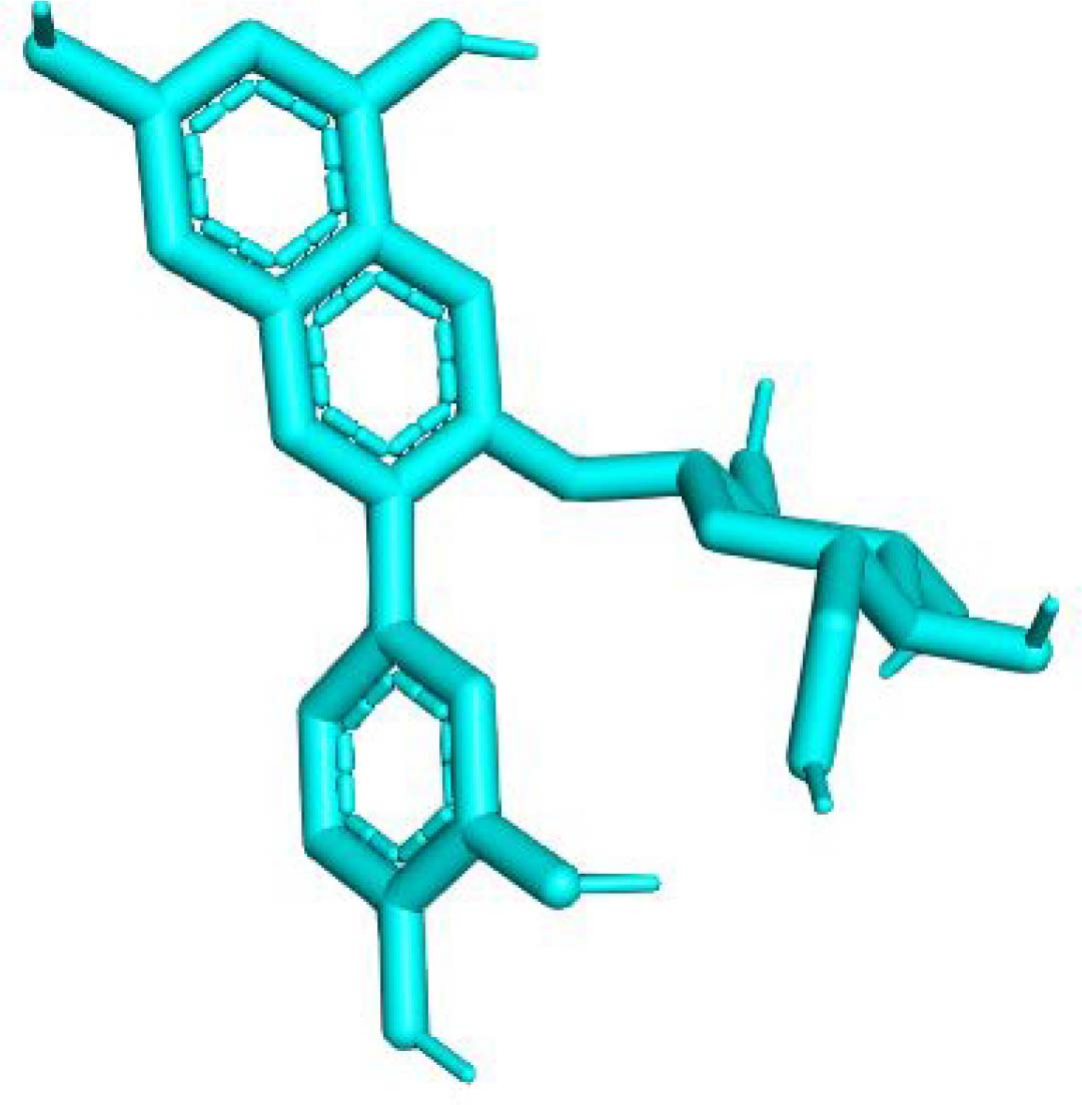 **cyanidin-3-O-glucoside**	−8.9	His80 Leu84 Thr87 Val95 Phe98 Val157 Lys321 Gln457	3.51 4.70 2.95 2.70; 5.16 2.33 4.47 3.10 3.09; 3.98	*** *** 2.95 2.70 2.33 *** 3.10 3.09; 3.98
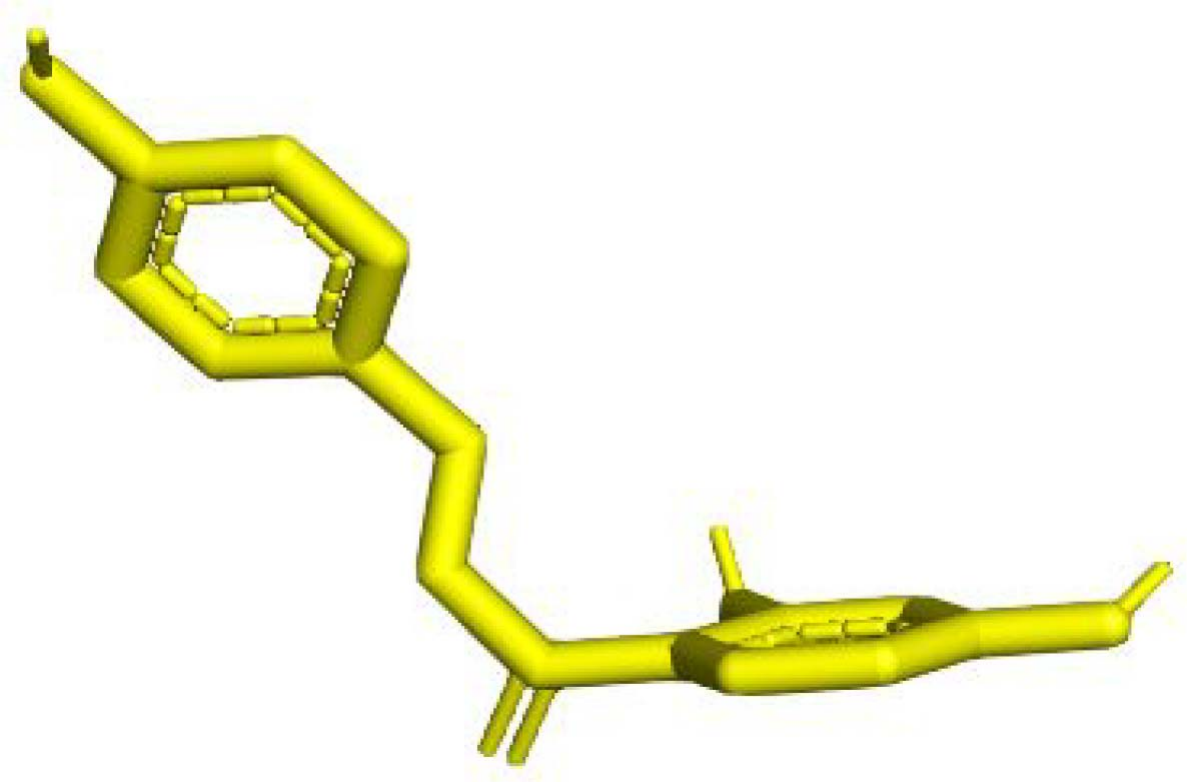 **isoliquiritigenin**	−8.9	Asn75 Leu84 Phe98 Glu99 Tyr290	2.99; 3.29 5.28 5.32 2.86 3.01; 3.69	2.99; 3.29 *** *** 2.86 3.01
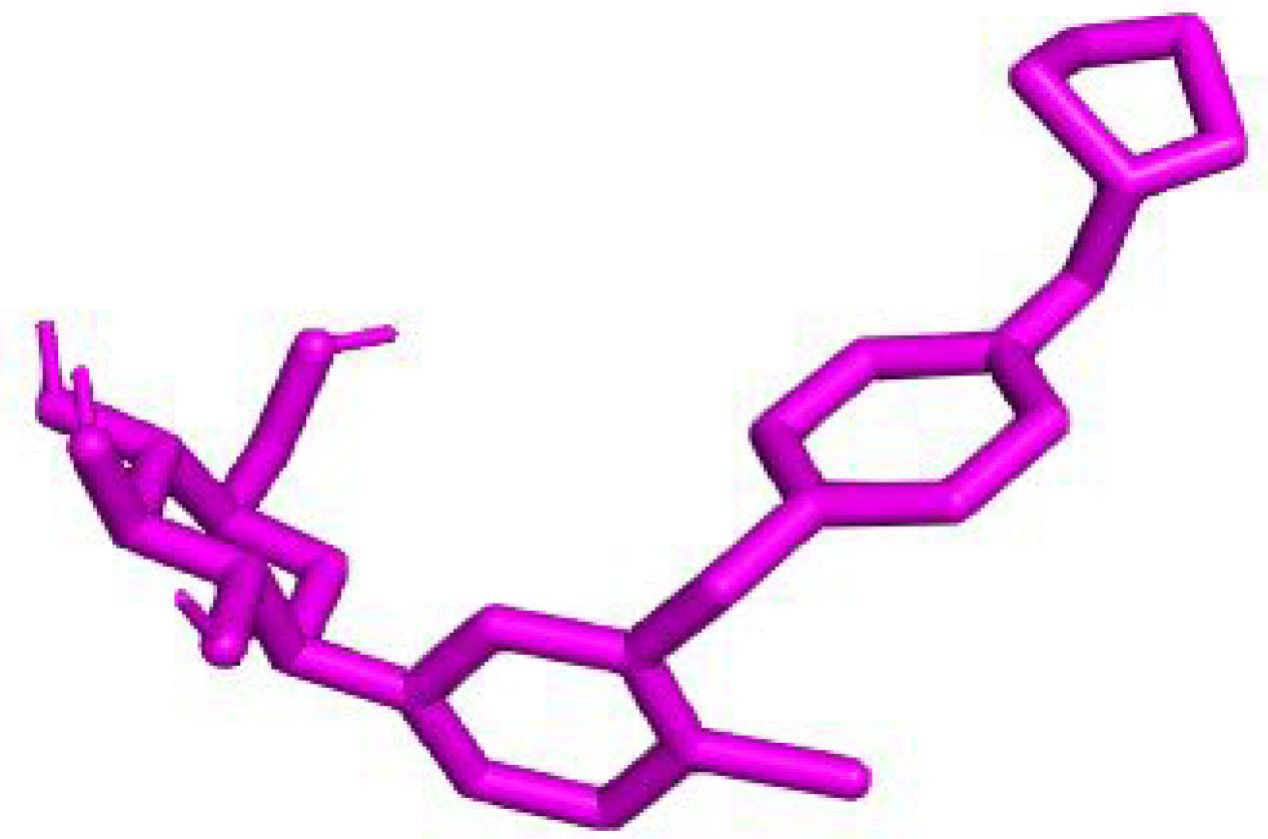 **Co-7VSI**	−10.9	Asn75 His80 Val95 Phe98 Glu99 Leu274 Tyr290 Lys321 Phe453 Asp454 Gln457 Tyr526	2.14 3.00; 3.49 5.11; 5.29 2.52 2.69 5.41 3.73 1.63 5.58 3.47 2.01 3.02	2.14 3.00 *** 2.52 2.69 *** *** *** *** 3.47 2.01 3.02

**Figure 6.**
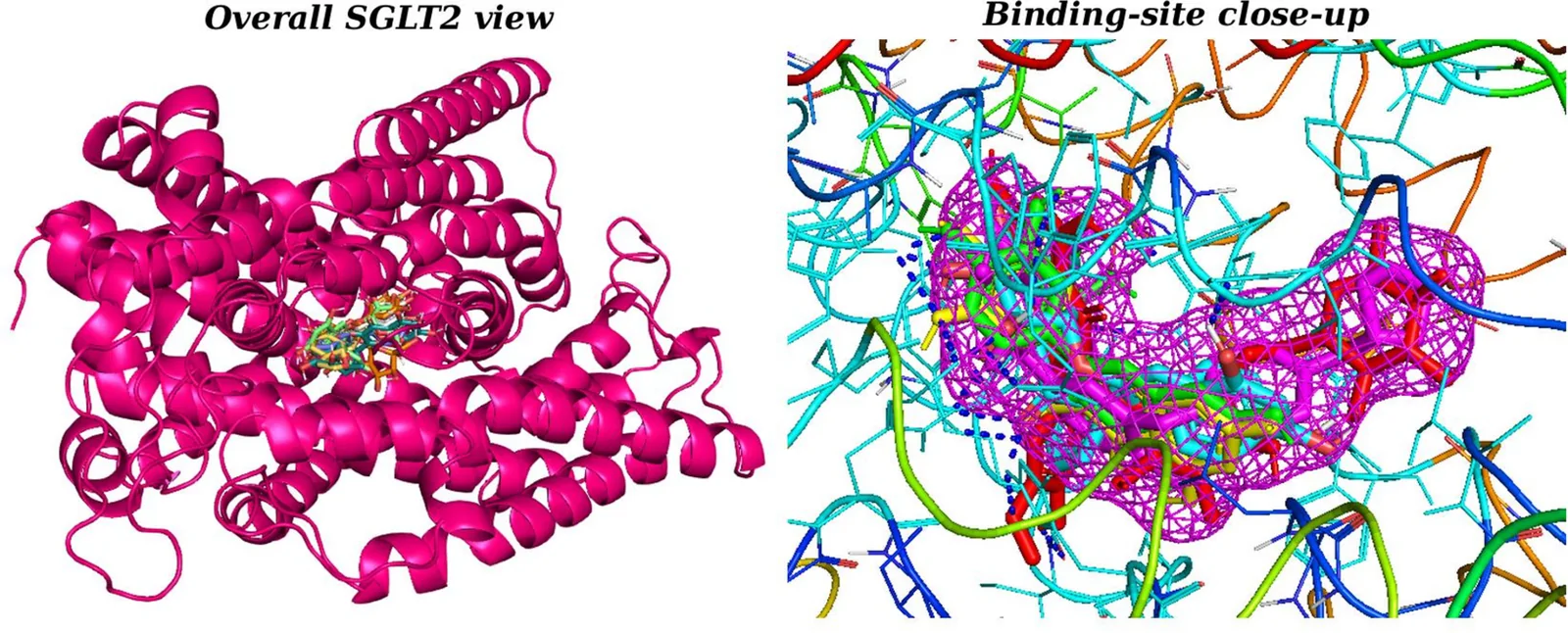
Superposition of the four docked phytochemicals with the co-crystallized inhibitor in SGLT2 (PDB 7VSI); zoomed view shows the common binding cavity occupied by all ligands.

**Figure 7.**
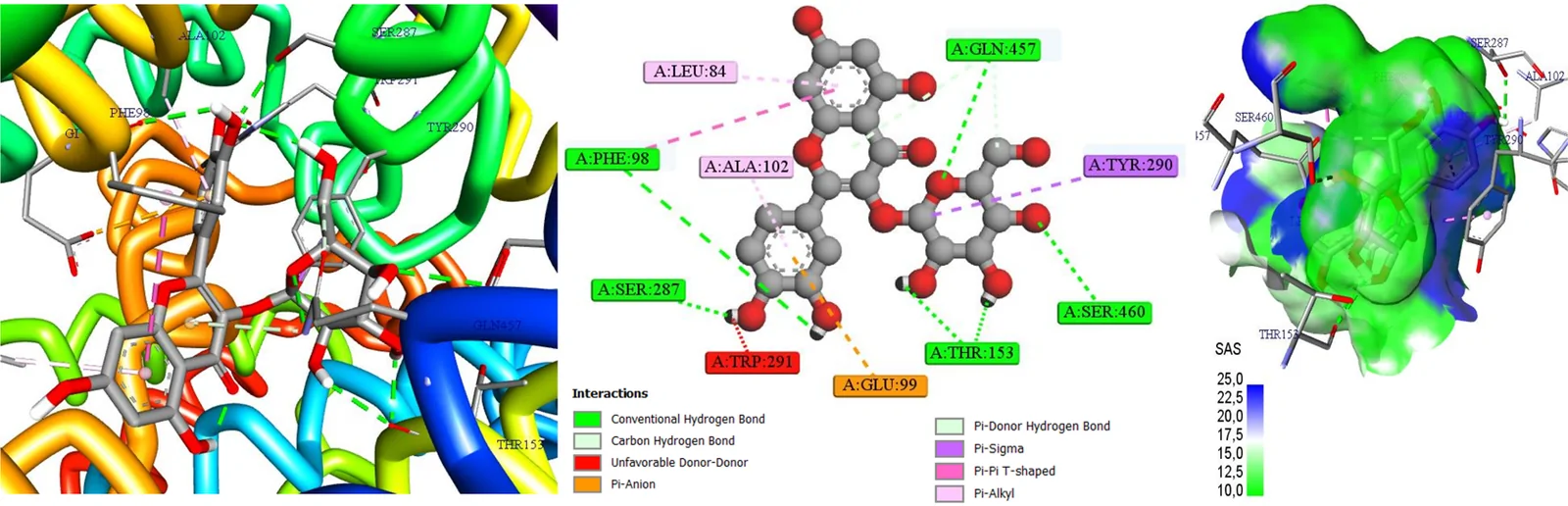
Two-dimensional/three-dimensional interaction map and SAS surface of hyperoside bound to SGLT2 (PDB 7VSI).

**Figure 8.**
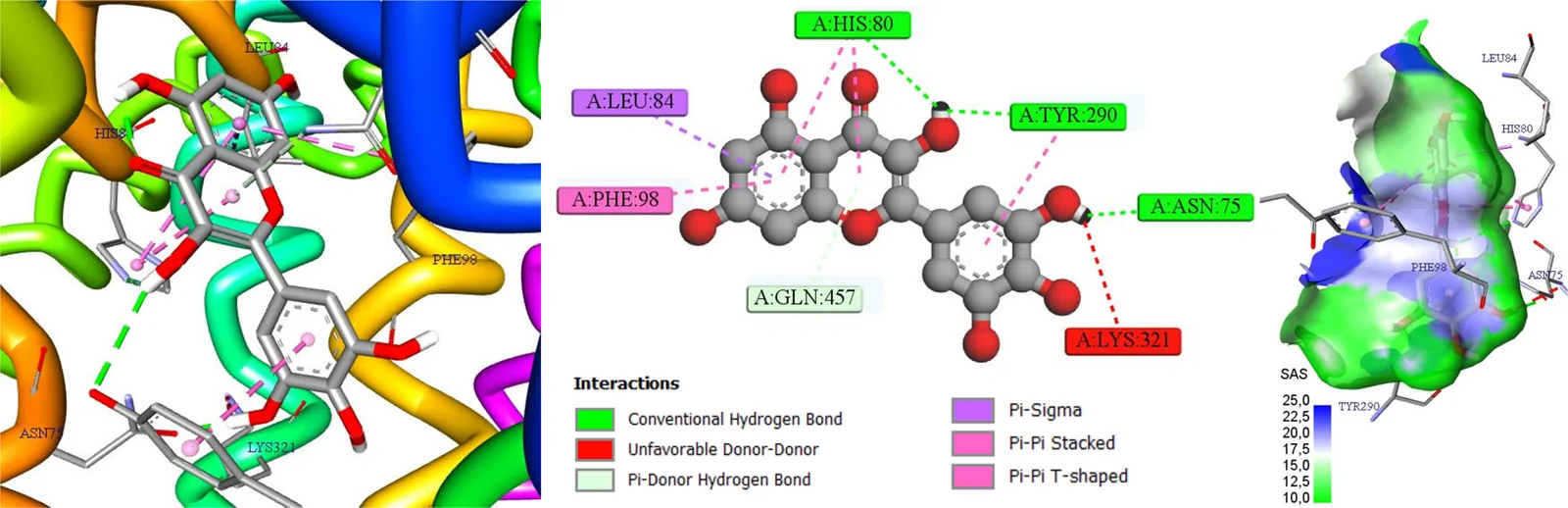
Two-dimensional/three-dimensional interaction map and SAS surface of myricetin bound to SGLT2 (PDB 7VSI).

**Figure 9.**
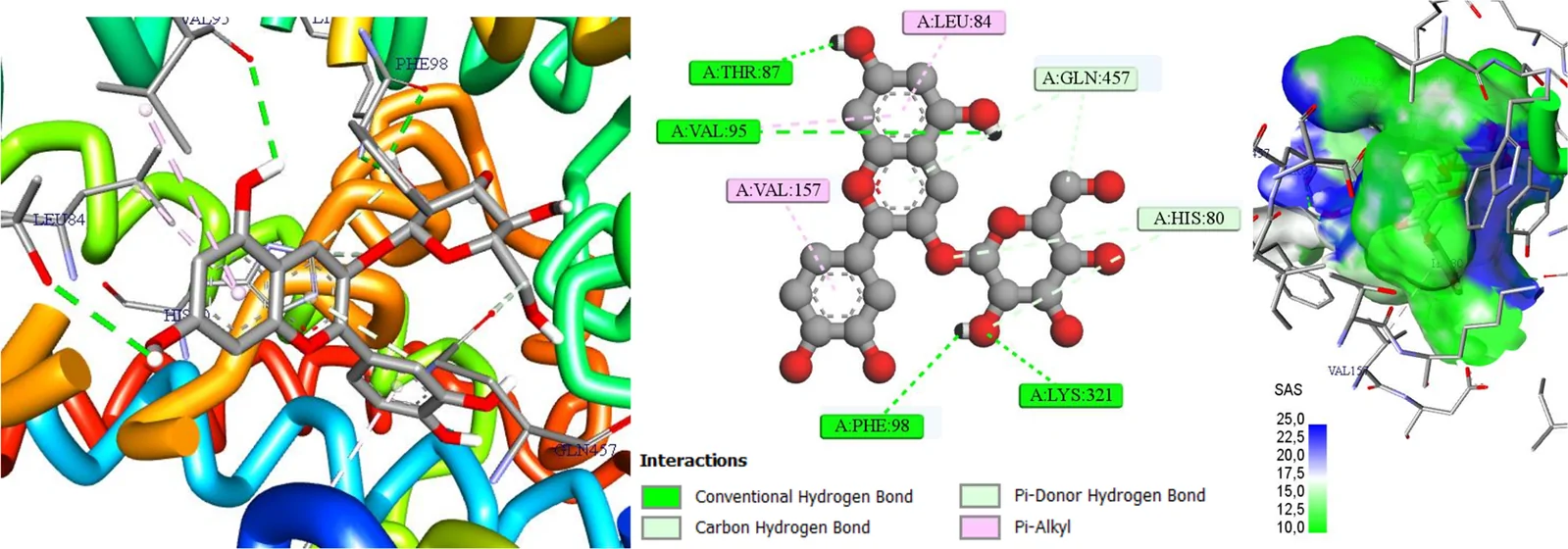
Two-dimensional/three-dimensional interaction map and SAS surface of cyanidin-3-O-glucoside bound to SGLT2 (PDB 7VSI).

**Figure 10.**
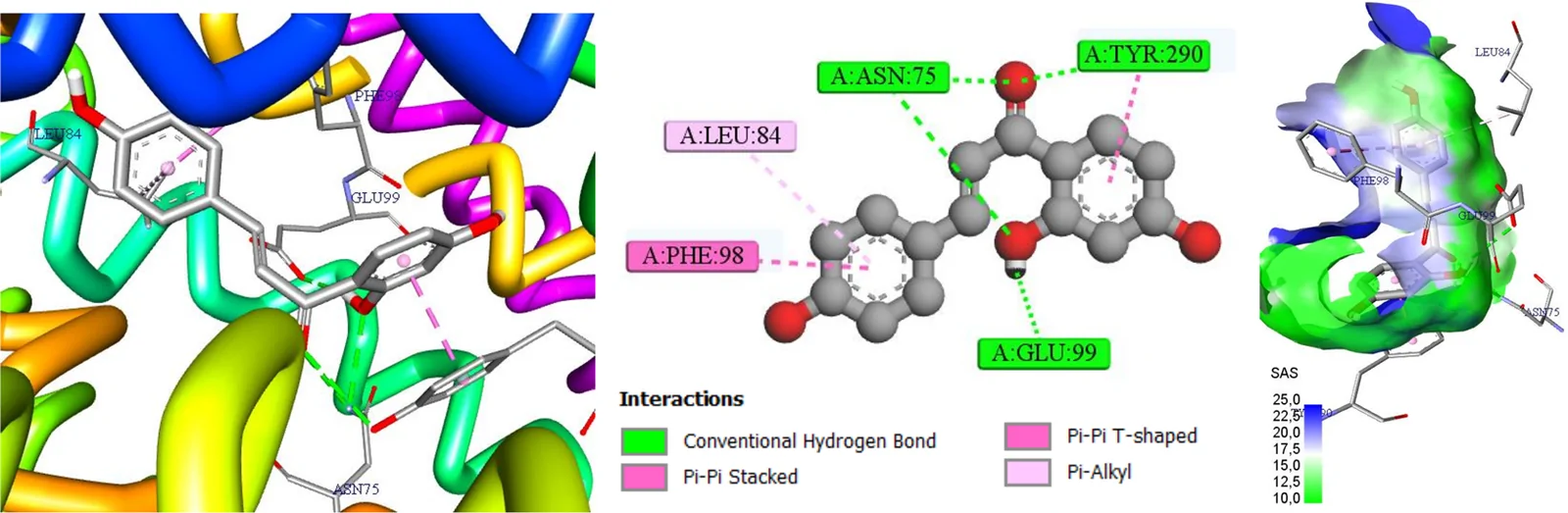
Two-dimensional/three-dimensional interaction map and SAS surface of isoliquiritigenin bound to SGLT2 (PDB 7VSI).

For Hyperoside-7VSI (figure 7), the two-dimensional/three-dimensional maps show six conventional hydrogen bonds: two with Thr153 and one each with Phe98, Ser287, Gln457 and Ser460. In addition, there are seven secondary contacts spanning *π*-*σ*, *π*-*π* T-shaped, *π*-donor H-bond, carbon-hydrogen bond, *π*-alkyl and *π*-anion types, which collectively clamp the chromophore within the aromatic subpocket, while the 3-O-glucoside chelates the polar cradle. One unfavourable donor–donor contact is flagged with Trp291, suggesting a minor local strain that MD relaxation may resolve. The small-angle scattering (SAS) surface analysis reported more than 85% of the pocket area in the green band 10.0–17.5, consistent with a well-packed, stable complex [46]. For myricetin-7VSI (figure 8), only three conventional hydrogen bonds were observed (His80, Tyr290, Asn75), complemented by five aromatic/hydrophobic contacts that include *π*-*π* stacked, *π*-*π* T-shaped, *π*-donor H-bond and *π*-*σ* interactions. An unfavourable donor–donor alert involves Lys321. The SAS readout shows approximately 50% of the surface within 10.0–17.5, indicating looser packing and a greater reliance on *π*-contacts than on multidentate hydrogen bonding.

For cyanidin-3-O-glucoside-7VSI (figure 9), the ligand forms four conventional hydrogen bonds (Thr87, Val95, Phe98, Lys321), together with six secondary contacts distributed among *π*-donor H-bond, carbon-hydrogen bond and *π*-alkyl types. The SAS profile shows more than 70% in the 10.0–17.5 band, pointing to a compact fit, where the glycone anchors the polar cradle and the flavylium core nestles against the aromatic surfaces. For Isoliquiritigenin-7VSI (figure 10), there were four conventional hydrogen bonds: two with Asn75 and one each with Tyr290 and Glu99. Three aromatic/hydrophobic contacts were detected (*π*-*π* T-shaped, *π*-alkyl, *π*-*π* stacked). Despite the open-chain enone, the SAS analysis indicated approximately 75% of the surface was in 10.0–17.5, suggesting acceptable packing aided by *π*-stacking but fewer directional polar anchors than the glycosides [47–49].

The glycosides (hyperoside and cyanidin-3-O-glucoside) displayed richer hydrogen-bond networks to the polar sugar-recognition cradle and larger green SAS fractions, which rationalized their tighter, more stable binding. The aglycones (myricetin and isoliquiritigenin) depend more on *π*-driven contacts; myricetin shows the sparsest SAS green fraction and an unfavourable donor–donor flag, consistent with a less optimally anchored complex, whereas isoliquiritigenin achieves reasonable packing through aromatic stacking despite fewer polar contacts. These features in figures 7–10 help nominate residues for targeted mutational or per-residue energy analysis in the next stage.

### 3.3. Membrane-embedded molecular dynamics analysis

Membrane-embedded MD analysis was used to determine whether the docked poses remained compatible with the lipid-coupled conformational behaviour of SGLT2. Four complementary descriptors were examined: ligand heavy-atom RMSD after fitting the trajectory to the protein backbone, protein backbone root mean square fluctuation (RMSF), the time-resolved number of direct protein–ligand hydrogen bonds and the protein radius of gyration (Rg) [50,51].

Figure 11A shows that myricetin retained the smallest pose deviation throughout most of the trajectory (approx. 0.05–0.15 nm), while cyanidin-3-O-glucoside remained within a narrow intermediate range (approx. 0.18–0.30 nm). Hyperoside underwent an early positional adjustment and then maintained a stable plateau near 0.40–0.48 nm, indicating accommodation in a persistent binding geometry rather than a progressive escape. By contrast, isoliquiritigenin displayed stepwise displacement after approximately 25 ns and a pronounced late increase to approximately 0.8–1.0 nm after 80 ns, consistent with a major binding-mode rearrangement or partial departure from the initial pocket pose [52].

**Figure 11.**
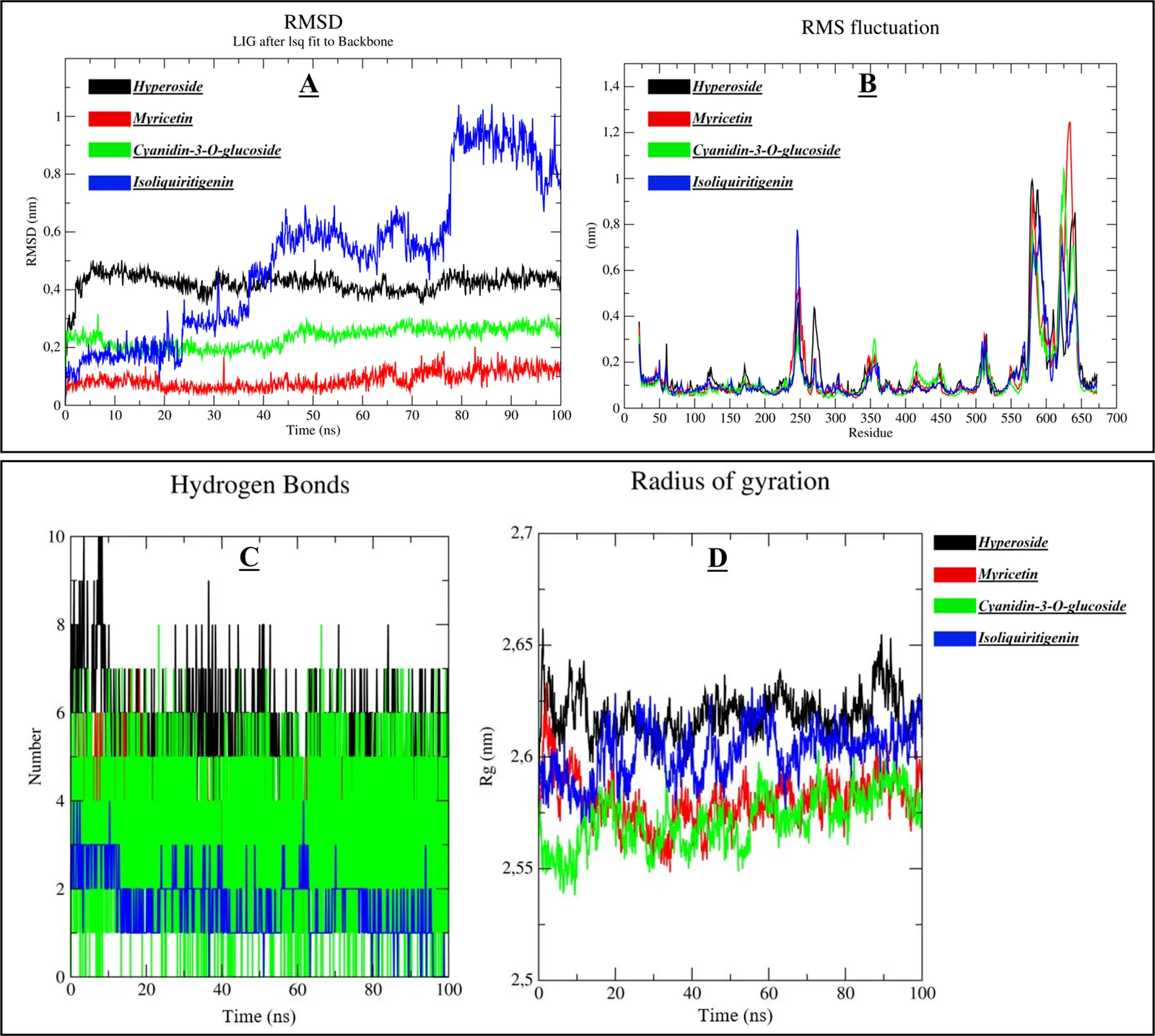
Stability of the membrane-embedded SGLT2–ligand complexes during 100 ns simulations. (A) Ligand heavy-atom RMSD after least-squares fitting to the protein backbone. (B) Protein backbone RMSF per residue. (C) Time-resolved number of direct protein–ligand hydrogen bonds. (D) Protein radius of gyration (Rg).

The backbone RMSF profiles in figure 11B remained low across most transmembrane helices, with the largest fluctuations confined to solvent-exposed loops, terminal regions and the reconstructed 571–641 segment and its flanking residues, which are spatially remote from the active site. The absence of a sustained increase in Rg (approx. 2.55–2.64 nm across all systems; figure 11D) argues against global unfolding and indicates preservation of the membrane-embedded transporter fold. Hydrogen-bond analysis (figure 11C) further showed the richest and most persistent polar networks for hyperoside and cyanidin-3-O-glucoside, an intermediate pattern for myricetin and the fewest contacts for isoliquiritigenin. Together, these results identify cyanidin-3-O-glucoside as a dynamically well-anchored glycoside, myricetin as a positionally stable aglycone and isoliquiritigenin as the least persistent ligand under the membrane conditions.

### 3.4. Molecular mechanics/Poisson–Boltzmann surface area binding-energy analysis

MM/PBSA analysis was used to compare the ensemble-averaged non-bonded interaction and continuum-solvation terms retained over the equilibrated 75–100 ns interval. Negative van der Waals (Δ*E*_vdW_) and electrostatic (Δ*E*_elec_) terms favour association, whereas the positive Poisson–Boltzmann contribution reflects polar desolvation; therefore, the reported Δ*G*bind values are relative binding-energy estimates rather than absolute free energies [53].

Across 100 snapshots, all four complexes yielded favourable mean Δ*G*_bind_ values (table 2). Cyanidin-3-O-glucoside was the most favourable complex (–33.70 ± 5.99 kcal mol^−1^), driven by the strongest electrostatic stabilization (–91.07 ± 6.84 kcal mol^−1^) together with substantial van der Waals interactions (–41.48 ± 3.82 kcal mol^−1^), despite the largest polar desolvation penalty (+103.69 ± 7.02 kcal mol^−1^). Hyperoside (–23.03 ± 4.67 kcal mol^−1^) and myricetin (–22.83 ± 3.87 kcal mol^−1^) were effectively comparable within their dispersion; both showed nearly identical van der Waals stabilization, whereas the stronger electrostatic term of hyperoside was largely offset by a larger Poisson–Boltzmann penalty. Isoliquiritigenin was the least favourable (–18.94 ± 2.96 kcal mol^−1^) because its weaker van der Waals and electrostatic terms were not compensated by its smaller solvation cost [54].

**Table 2.** MM/PBSA energy components and relative binding-energy estimates (kcal mol^−1^; mean ± s.d.) for SGLT2–ligand complexes, calculated from 100 snapshots uniformly sampled over 75–100 ns. Configurational entropy and explicit membrane contributions were not included.

compounds	Δ*E*_vdW_	Δ*E*_elec_	Δ*G*_gas_	Δ*E*_PB_	Δ*E*_npolar_	Δ*G*_solv_	Δ*G*_bind_
**hyperoside**	–42.85 ± 3.23	–57.95 ± 7.65	–100.80 ± 6.64	+82.51 ± 6.96	–4.74 ± 0.10	+77.78 ± 6.98	–23.03 ± 4.67
**myricetin**	–42.91 ± 2.58	–31.28 ± 5.39	–74.18 ± 4.34	+55.01 ± 3.98	–3.66 ± 0.06	+51.35 ± 4.00	–22.83 ± 3.87
**cyanidin-3-O-glucoside**	–41.48 ± 3.82	–91.07 ± 6.84	–132.55 ± 6.75	+103.69 ± 7.02	–4.85 ± 0.07	+98.85 ± 7.01	–33.70 ± 5.99
**isoliquiritigenin**	–29.12 ± 2.43	–13.86 ± 4.66	–42.98 ± 4.40	+27.52 ± 4.96	–3.48 ± 0.09	+24.04 ± 4.94	–18.94 ± 2.96

The resulting order is cyanidin-3-O-glucoside > hyperoside ≈ myricetin > isoliquiritigenin. This ranking is consistent with the persistent membrane-trajectory behaviour of cyanidin-3-O-glucoside and the marked late drift of isoliquiritigenin; however, the overlap between the hyperoside and myricetin distributions does not support a strict energetic separation between those two complexes.

### 3.5. Principal component and free-energy landscape analysis

PCA was applied to the equilibrated 75–100 ns interval to reduce the collective protein motions to PC1 and PC2 and the corresponding population distributions were converted into free-energy landscapes. These maps describe the conformational organization and interconversion among the sampled substates; they do not independently quantify ligand affinity [55].

Figure 12 shows a distinct conformational signature for each membrane-embedded complex. Hyperoside samples a narrow, elongated low-energy trough, indicating a coherent, dominant, collective pathway with limited excursions orthogonal to that motion. Myricetin occupied several separated basins despite its low ligand RMSD, suggesting exchange among protein-level substates without evidence of substantial ligand displacement. Cyanidin-3-O-glucoside explored multiple broad but connected minima, consistent with an anchored ligand accommodated by adaptive rearrangements of the surrounding transporter. Isoliquiritigenin exhibited the most branched and heterogeneous landscape, with several accessible minima distributed across the PC plane; this organization agrees with its stepwise RMSD increase and weaker MM/PBSA estimate.

**Figure 12.**
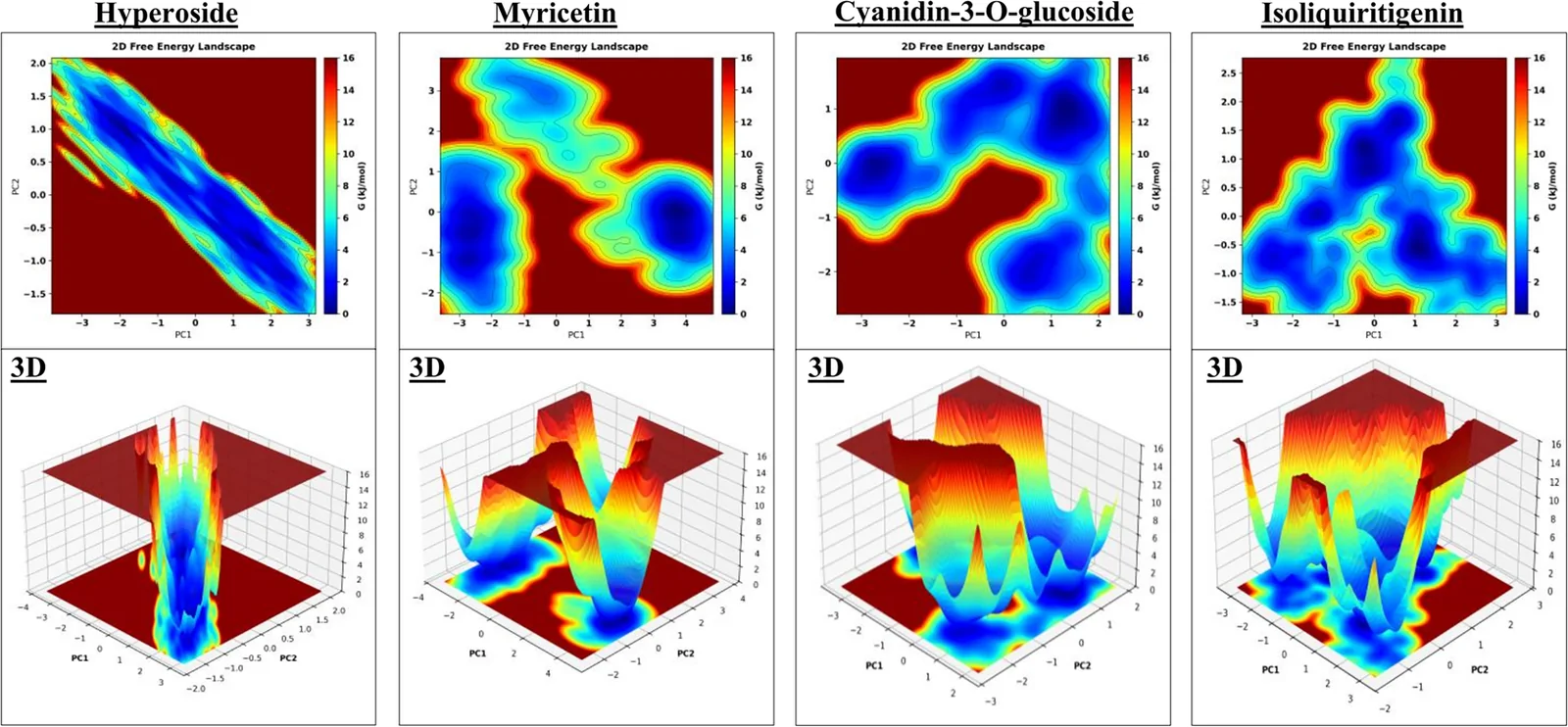
PCA-derived two-dimensional and three-dimensional free-energy landscapes of the membrane-embedded SGLT2 complexes with hyperoside, myricetin, cyanidin-3-O-glucoside and isoliquiritigenin, projected onto PC1 and PC2 using the 75–100 ns interval. Each landscape was normalized independently.

Because each landscape was normalized independently, basin colours or apparent depths could not be compared across complexes as absolute binding-energy measures. Therefore, the PCA analysis complements, rather than replaces, the ligand RMSD and MM/PBSA results: cyanidin-3-O-glucoside combines favourable energetics with adaptive conformational sampling, whereas isoliquiritigenin shows both pose instability and a more heterogeneous ensemble.

### 3.6. Density functional theory analysis

#### 3.6.1. Frontier orbitals and conceptual-density-functional-theory descriptors

DFT descriptors were evaluated for hyperoside, myricetin and cyanidin-3-O-glucoside to compare ligand-intrinsic electronic rigidity, polarizability and charge-redistribution propensity. Frontier-orbital and conceptual-DFT quantities were used here as qualitative electronic descriptors and not as substitutes for membrane-trajectory or binding-energy analysis [56,57].

Table 3 shows that cyanidin-3-O-glucoside has the smallest HOMO–LUMO gap (2.67 eV), lowest hardness (1.34 eV) and highest softness (0.37 eV^−1^), together with a strongly negative chemical potential (–7.14 eV) and the largest electrophilicity index (19.10 eV). Therefore, it is the most electronically responsive member of the three-compound DFT subset. Hyperoside and myricetin display nearly identical gaps (4.11 and 4.13 eV), hardness values (2.06 and 2.07 eV) and softness values (0.24 eV^−1^ for both), while their chemical potentials (–3.59 and –3.44 eV) and electrophilicity indices (3.12 and 2.86 eV) were also similar. These values indicate comparable intrinsic electronic rigidity for the two flavonols.

**Table 3.** Frontier-orbital energies and conceptual DFT descriptors for hyperoside, myricetin and cyanidin-3-O-glucoside. Energies, hardness, chemical potential and electrophilicity are reported in eV and softness is reported in eV^−1^.

descriptor	symbol	hyperoside	myricetin	cyanidin-3-O-glucoside
HOMO energy	*E*_HOMO_	–5.64	–5.50	–8.48
LUMO energy	*E*_LUMO_	–1.54	–1.37	–5.80
energy gap (HOMO-LUMO)	*E*gap	4.11	4.13	2.67
hardness	*η*	2.06	2.07	1.34
softness	*S* (eV^−1^)	0.24	0.24	0.37
chemical potential	*μ*	–3.59	–3.44	–7.14
electrophilicity index	*ω*	3.12	2.86	19.10

**Equations used:**

*E*gap = *E*_LUMO_ – *E*_HOMO,_

*η* (hardness) = *E*gap / 2,

*S* (softness) = 1 / (2*η*) [units: eV^−1^],

*μ* (chemical potential) = (*E*_HOMO_ + *E*_LUMO_) / 2,

*ω* (electrophilicity) = *μ*² / (2*η*).

The frontier-orbital maps in figure 13 support this distinction. Myricetin exhibits orbital density distributed across its planar flavonol core, whereas hyperoside combines a conjugated aromatic region with a glycosidic domain that is electronically less integrated with the *π* framework. Cyanidin-3-O-glucoside showed lower-lying frontier orbitals and extended density over the chromophore, consistent with its greater charge-redistribution propensity. This electronic responsiveness provides a qualitative rationale for the strong electrostatic MM/PBSA term of cyanidin-3-O-glucoside, while the stable membrane behaviour of myricetin demonstrates that geometric complementarity and local contacts can also sustain binding in the absence of an unusually small frontier-orbital gap.

**Figure 13.**
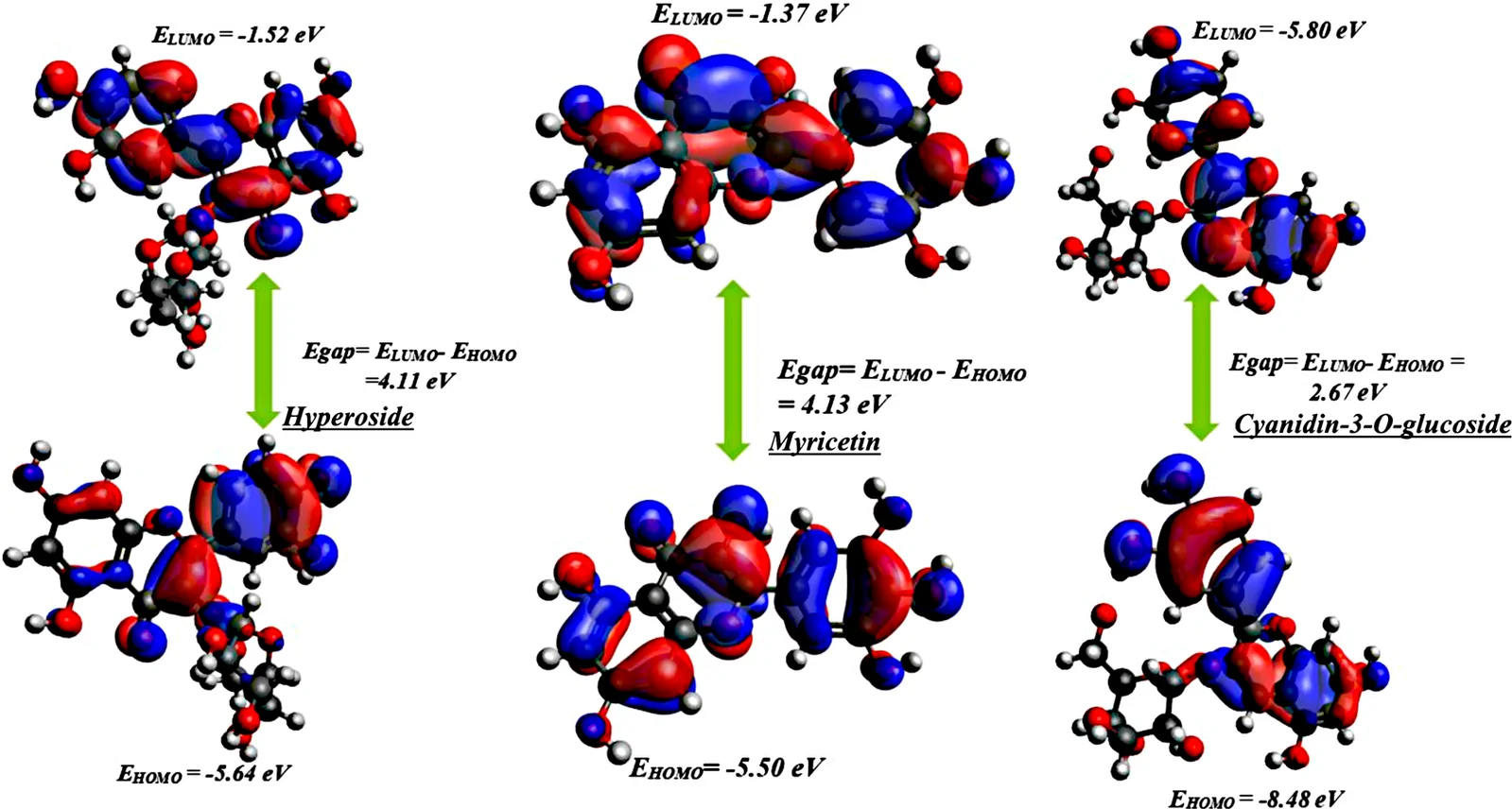
Frontier-orbital isosurfaces (HOMO and LUMO) and HOMO–LUMO energy gaps for hyperoside, myricetin and cyanidin-3-O-glucoside at the B3LYP-D3(BJ)/def2-SVP level.

These gas-phase descriptors should not be interpreted as quantitative predictors of affinity in a hydrated, membrane-embedded transporter, where microspeciation, desolvation, structured water, lipid electrostatics and conformational gating contribute to recognition. Therefore, their value is mechanistic and comparative when interpreted together with docking, membrane MD, MM/PBSA and PCA [31,58].

#### 3.6.2. Real-space electronic landscape analysis

MEP, ELF and LOL analyses complement the scalar DFT descriptors by locating the electrostatic donor/acceptor regions, localized lone-pair domains and pathways of *π*-electron delocalization on each ligand. The maps in figures 14 and 15 were generated with identical visualization settings, allowing direct qualitative comparison among hyperoside, myricetin and cyanidin-3-O-glucoside [59].

**Figure 14.**
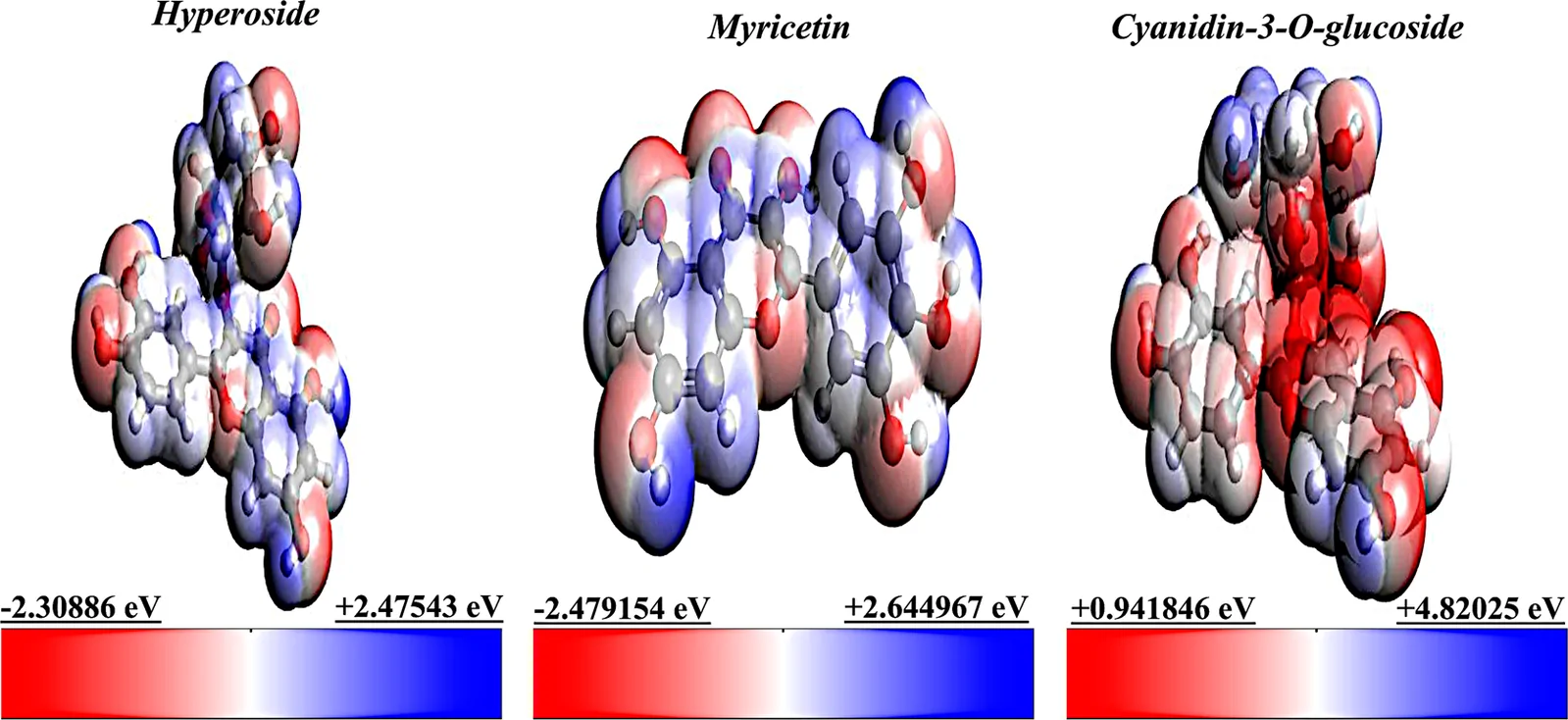
MEP (eV) mapped onto the electron-density surfaces of hyperoside, myricetin and cyanidin-3-O-glucoside using identical display parameters.

**Figure 15.**
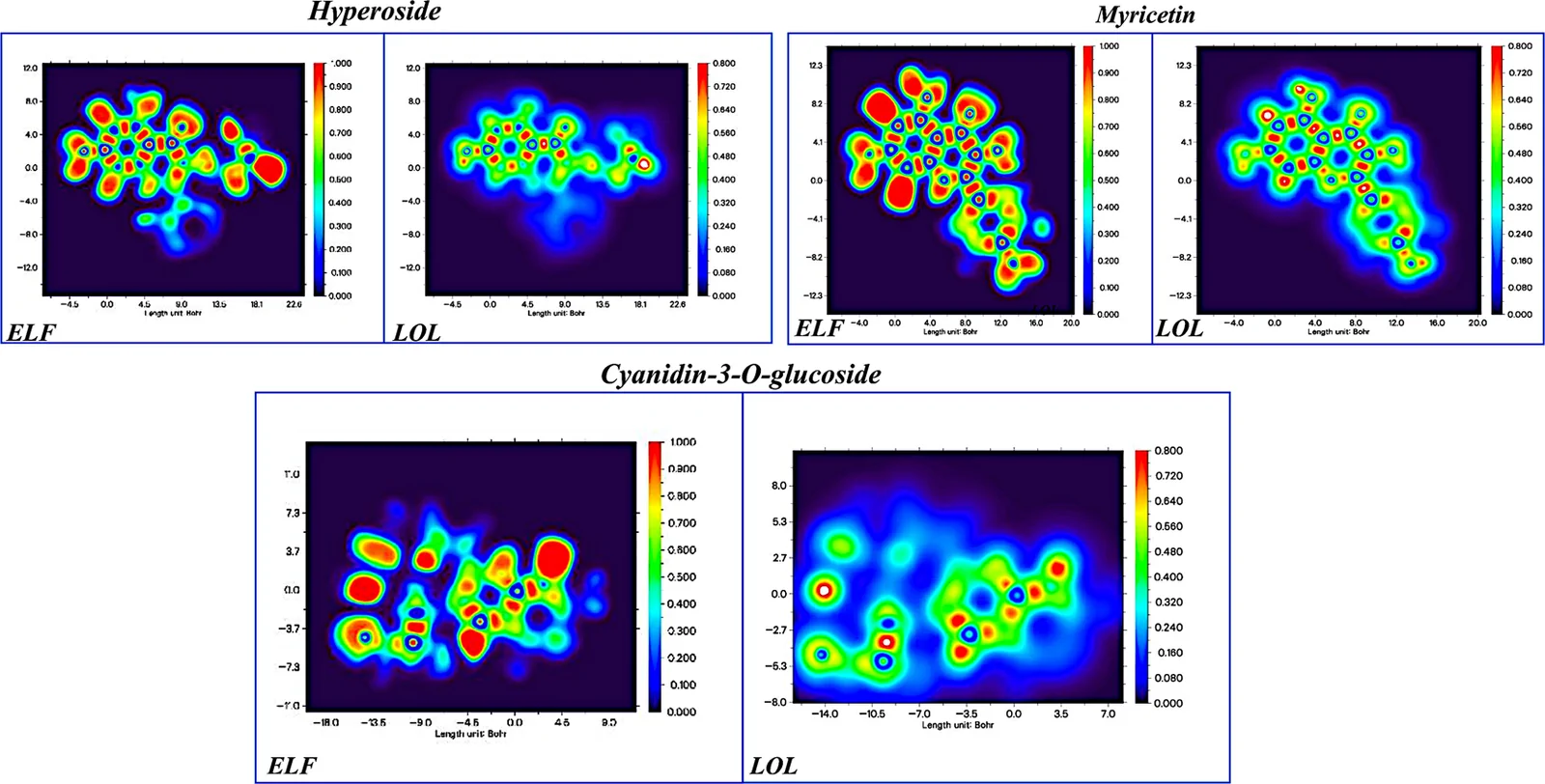
Electron localization function (ELF) and localized orbital locator (LOL) maps for hyperoside, myricetin and cyanidin-3-O-glucoside, generated using identical grids and visualization settings.

In figure 14, hyperoside and myricetin display amphipolar MEP surfaces spanning –2.31 to +2.48 eV and –2.48 to +2.64 eV, respectively. Their negative regions are concentrated around phenolic, carbonyl and glycosidic oxygen atoms, whereas positive regions occur around hydroxyl hydrogens, producing complementary donor–acceptor patterns. Myricetin presents this pattern across a comparatively planar aglycone surface, while hyperoside distributes it over both the flavonol and sugar domains. Cyanidin-3-O-glucoside exhibited a markedly shifted positive MEP range (+0.94 to +4.82 eV), revealing a distinct electrostatic surface that is consistent with its lower-lying frontier levels and pronounced electrostatic contribution in the MM/PBSA analysis.

The ELF maps in figure 15 show strongly localized basins around oxygen lone pairs and covalent** *σ* bonds for all three ligands. The corresponding LOL maps distinguish their conjugation patterns: myricetin maintains a continuous delocalized pathway across the planar flavonol core; hyperoside retains delocalization mainly within the aromatic chromophore, with interruption across the glycosidic linkage; and cyanidin-3-O-glucoside combines an extended delocalized chromophore with localized sugar-based electron pairs. Collectively, figures 14 and 15 indicate that cyanidin-3-O-glucoside is the most electronically responsive ligand, whereas hyperoside and myricetin offer more localized but chemically complementary donor–acceptor surfaces [60,61].

### 3.7. Integrated mechanistic interpretation and study limitations

Related natural-product studies have established that oxygenated flavonoids and glycosides can inhibit SGLT2, providing external support for the chemical space examined here [7,29,30]. The present compounds were not selected as a homologous optimization series; rather, they constitute an orthogonal mechanistic panel that separates glycosylation, aglycone planarity, chromophore electronics and chalcone flexibility. This design enables the observed polar-cradle/aromatic-shelf recognition pattern to be tested across chemically distinct scaffolds [7,54,62].

For the exact ligands studied, current experimental evidence remains indirect with respect to hSGLT2: hyperoside has shown glucose-lowering and *β*-cell-protective effects [31,32], myricetin has demonstrated insulinotropic and anti-diabetic activity [63,64], cyanidin-3-O-glucoside improves insulin signaling and glucose handling [58,65–67] and isoliquiritigenin modulates glucose-homeostasis pathways in cellular and animal models [68,69]. These reports support biological plausibility but do not establish direct SGLT2 inhibition.

Mechanistically, the integrated results refined the docking interpretation without converting the correlation into proof of modulation. The docking poses placed the ligands within the canonical polar/aromatic cavity and identified recurrent contacts involving residues such as Phe98, Glu99, Tyr290, Lys321 and Gln457. In the membrane simulations, cyanidin-3-O-glucoside retained a stable pose and the most favourable MM/PBSA estimate, hyperoside and myricetin formed an energetically overlapping intermediate group through different balances of electrostatics and desolvation and isoliquiritigenin showed both the weakest energy estimate and substantial late pose drift. The DFT analysis provides a ligand-intrinsic electronic context but cannot quantify transporter affinity [37,70–72].

The study remains hypothesis-generating. The 100 ns trajectories were not independently replicated, conventional MM/PBSA omitted configurational entropy and the heterogeneous membrane dielectric environment, and the DFT calculations described isolated-ligand electronic properties. Direct uptake or inhibition assays in hSGLT2-expressing cells, counter-screening against hSGLT1, targeted mutagenesis of the predicted pocket residues and replicate membrane simulations are therefore required to establish potency, selectivity and mechanism [4,68].

## 4. Conclusion

By reconstructing the unresolved SGLT2 segment and embedding each SGLT2–ligand complex in an explicit POPC bilayer, this study provides a physiologically better-grounded multi-scale comparison of the four natural chemotypes. Cyanidin-3-O-glucoside showed the most favourable relative MM/PBSA profile, maintained a stable pose throughout the membrane trajectory and displayed the greatest electronic responsiveness in the DFT analysis. Hyperoside and myricetin formed an energetically overlapping intermediate group but achieved stability through distinct mechanisms: hyperoside combined a persistent polar interaction network with aromatic packing, whereas myricetin exhibited the lowest ligand RMSD despite weaker electrostatic stabilization. Isoliquiritigenin underwent substantial late-stage pose rearrangement and showed the least favourable MM/PBSA profile.

Collectively, these findings support a design principle in which polar anchoring within the sugar-recognition cradle must be balanced with aromatic complementarity and restricted conformational flexibility. Nevertheless, the resulting ranking should be regarded as mechanistic prioritization rather than evidence of pharmacological inhibition. Additional independent membrane trajectories, membrane-aware free-energy approaches, hSGLT2/hSGLT1 functional assays and residue-directed experimental validation are required before these compounds can be considered selective SGLT2 modulators.

## Data Availability

All data, input files, analysis scripts and materials required to reproduce this study, including the POPC membrane MD simulations, are freely available at https://github.com/khibech/POPC.SGLT2 and have been archived in the Zenodo repository at [73].
